# Ongoing niche differentiation under high gene flow in a polymorphic brackish water threespine stickleback (*Gasterosteus aculeatus*) population

**DOI:** 10.1186/s12862-018-1128-y

**Published:** 2018-02-05

**Authors:** Kjartan Østbye, Annette Taugbøl, Mark Ravinet, Chris Harrod, Ruben Alexander Pettersen, Louis Bernatchez, Leif Asbjørn Vøllestad

**Affiliations:** 1 Department of Forestry and Wildlife Management, Inland Norway University of Applied Sciences, Campus Evenstad, NO2418 Elverum, Norway; 2 Department of Biosciences, Centre for Ecological and Evolutionary Synthesis (CEES), University of Oslo, Po. Box 1066, Blindern, N-0316 Oslo, Norway; 3 Norwegian Institute for nature research (NINA), Fakkelgården, 2624 Lillehammer, Norway; 4 Department of Physiological Ecology, Max Planck Institute for Limnology, Postfach 165, D-24302 Plön, Germany; 5 Universidad de Antofagasta, Fish and Stable Isotope Ecology Laboratory, Instituto de Ciencias Naturales Alexander von Humbolt, Avenida Angamos, 601 Antofagasta, Chile; 6 Department of Biology, Université Laval, Pavillon Charles-Eugène-Marchand 1030, Avenue de la Medecine, Quebec, G1V 0A6 Canada

**Keywords:** Adaptation, *Ectodysplasin*, Evolution, Gill raker, Natural selection, Panmixia, Stable isotope analyses, *Stn382*, *Theristina gasterostei*, *Trematoda* spp

## Abstract

**Background:**

Marine threespine sticklebacks colonized and adapted to brackish and freshwater environments since the last Pleistocene glacial. Throughout the Holarctic, three lateral plate morphs are observed; the low, partial and completely plated morph. We test if the three plate morphs in the brackish water Lake Engervann, Norway, differ in body size, trophic morphology (gill raker number and length), niche (stable isotopes; δ^15^N, δ^13^C, and parasites (*Theristina gasterostei*, *Trematoda* spp.)), genetic structure (microsatellites) and the lateral-plate encoding *Stn382* (*Ectodysplasin*) gene. We examine differences temporally (autumn 2006/spring 2007) and spatially (upper/lower sections of the lake – reflecting low versus high salinity).

**Results:**

All morphs belonged to one gene pool. The complete morph was larger than the low plated, with the partial morph intermediate. The number of lateral plates ranged 8–71, with means of 64.2 for complete, 40.3 for partial, and 14.9 for low plated morph. Stickleback δ^15^N was higher in the lower lake section, while δ^13^C was higher in the upper section. Stickleback isotopic values were greater in autumn. The low plated morph had larger variances in δ^15^N and δ^13^C than the other morphs. Sticklebacks in the upper section had more *T. gasterostei* than in the lower section which had more *Trematoda* spp. Sticklebacks had less *T. gasterostei*, but more *Trematoda* spp. in autumn than spring. Sticklebacks with few and short rakers had more *T. gasterostei*, while sticklebacks with longer rakers had more *Trematoda*. spp. Stickleback with higher δ^15^N values had more *T. gasterostei,* while sticklebacks with higher δ^15^N and δ^13^C values had more *Trematoda* spp. The low plated morph had fewer *Trematoda* spp. than other morphs.

**Conclusions:**

Trait-ecology associations may imply that the three lateral plate morphs in the brackish water lagoon of Lake Engervann are experiencing ongoing divergent selection for niche and migratory life history strategies under high gene flow. As such, the brackish water zone may generally act as a generator of genomic diversity to be selected upon in the different environments where threespine sticklebacks can live.

**Electronic supplementary material:**

The online version of this article (10.1186/s12862-018-1128-y) contains supplementary material, which is available to authorized users.

## Background

Within most populations some individuals tend to be better able to disperse and successfully colonize new environments than others. Are such individuals genetically pre-adapted, environmentally cued, or simply a random draw of the population? This is a relevant question for the threespine stickleback, *Gasterosteus aculeatus*, a highly adaptable euryhaline species commonly observed in salt water, brackish water and fresh water throughout the Holarctic [1–3]. Although originally marine, parallel freshwater colonization has occurred following the last Pleistocene deglaciation [1, 4–6]. Sticklebacks have been studied in great detail in freshwater systems, while less is known regarding the very early steps preceding freshwater colonization. As such, studying sticklebacks in brackish water where gene flow from both marine ancestors and freshwater populations occur may increase our understanding of how divergent multifarious adaptive processes act under gene flow.

Threespine stickleback are morphologically diverse, for example there is extensive variation in the number of lateral plates. Here, three nominal lateral plate morphs are recognized [7, 8]; (1) a completely plated morph with a full cover of lateral plates along the body flank most commonly associated with salt and brackish water, (2) a partially plated morph with a reduced lateral plate cover along the body flank, but with a fully or partly developed keel on the tail, mostly in brackish water and fresh water, and (3) a low plated morph with only a few anterior lateral plates along the body lacking a keel, dominating in fresh water. In addition, a rare fourth morph lacking all lateral plates is only found in a few freshwater lakes [9]. Strong directional selection for loss of lateral plates has occurred during freshwater colonization, causing shifts in mean phenotype within a few generations [10]. As such, there appears to be a directional pattern of decreased number of lateral plates linked with salinity regimes.

At the Holarctic scale, lateral plate reduction in threespine stickleback has occurred repeatedly and independently as freshwater rivers and lakes were colonized after the last glacial [1]. Rapid adaptive loss of armor plates appears to be due to selection on the ancestral pool of standing genetic variation in the ocean [5, 11]. Genetic studies have shown that Ectodysplasin (*Eda*) on chromosome IV is a major bi-allelic locus for lateral plate development, with low (aa), partial (Aa) and completely plated morphs (AA). Additional QTLs also appear to play a role in determining lateral plate number within morphs [5]. The frequency of the *Eda* low allele (*a*) has been estimated for marine and anadromous threespine stickleback populations and range between 0.0–19.2% (see overview of studies presented in Bell et al. [12]). After colonization, an increase in the low plated morph implies strong selection on the *Eda* gene and/or other traits in the haplotype block [10–16]. Based on experimental crosses, and field surveys, the *Eda* locus can explain up to 70% of the variation in lateral plate number and is associated with lateral plate morphs in geographically diverse populations [5, 13, 14, 16–19].

Since stickleback plate morphs are associated with different salinity environments, an important question is whether the different plate morphs have other specific traits that potentially “pre-adapt” them to e.g. freshwater colonization. An experiment showed that offspring of partial- and low plated sticklebacks grew equally well as completely plated morphs when raised in salt water, but outcompeted the complete morph in fresh water [20]. In a common garden experiment using replicate semi-natural ponds in fresh water, the “low plate” *a* allele individuals had a higher juvenile growth rate and a higher overwinter survival while the *“*complete plate*” A* allele individuals caught up in size at sexual maturation [12]. Barret et al. [21] further showed that the low plated fish explored both salt and freshwater habitats regardless of being acclimated to salt or fresh water, while the completely plated fish preferred the environment they were acclimatized in. Furthermore, Greenwood et al. [22] showed that *Eda* partly explained schooling behaviour in freshwater benthic and marine pelagic threespine stickleback. A recent study by Robertson et al. [23] found an association between the *Eda* haplotype block and the relative expression of transcripts of immune system genes. In that study, *Eda* genotypes in F_2_ from an experimental cross of one freshwater lowplated and one marine completely plated fish where exposed to fresh and marine water. In that experiment, the *aa* low plated morphs had a lower growth rate than the faster growing *AA* completely plated fish in both water conditions, The lowplated morph also had a higher parasite load. These results imply that sticklebacks with a lowplated *aa* genotype may have a different and more explorative foraging behaviour, as well as potentially also different immune system genes compared to the two other *Eda* genotypes. Thus, *Eda* genotypes may be in linkage with genes underlying other adaptive traits important for colonization.

The efficiency of natural selection under gene flow remains an intriguing question in evolutionary biology (e.g. [24]). Under low levels of gene flow, populations may diverge due to local selection pressures, while under higher levels of gene flow from populations adapted to divergent environments local adaptations may be swamped by gene flow [25]. Many studies on sticklebacks have targeted freshwater populations and analyzed adaptive diversification in various habitat types (limnetic-benthic / lake-stream) (e.g. [1, 24]. In comparison, we know less about behaviour, life history, ecology and genetic structure in the brackish water zone sticklebacks (but see [16, 17, 26–31]. In brackish water, immigrants from fresh water and salt water may generate a zone of high genotypic and phenotypic diversity. Hybridization and genomic introgression between freshwater and saltwater adapted sticklebacks (as well as among plate morphs) could produce novel adaptive genetic combinations in this zone. Thus, sticklebacks of a specific genomic variant for a successful freshwater colonization may exist in brackish water being more prone to colonize fresh water when new opportunities arise. Depending upon the geographical area, postglacial lakes likely went through a temporal increasing isolation from the ocean due to isostatic rebound resulting in a variable timeframe of brackish water influence. Studying brackish water zones may be important for our general understanding of post-glacial colonization success of stickleback in the different salinity environments.

The main aim of this study was to describe niche occupation and associated morphological traits in three sympatric lateral plate morphs of threespine stickleback in the brackish water Lake Engervann, Norway. Here, we tested if morphs differed in body size, trophic morphology (gillraker number and raker length), and niche occupation (stable isotopes and parasite load). Microsatellites were used to test if morphs belonged to different genetic populations. We further tested for associations in niche occupation and trophic morphology in the three stickleback morphs to indirectly search for trait combinations related to the three underlying *Eda* genotypes. Studying the threespine stickleback in the brackish water zone is important for our understanding of how adaptation may proceed in such zone of high gene flow, as well as how the brackish water population may act as a facilitator of rapid adaptive radiation by likely harboring genomic combinations from divergent environments.

## Methods

### Study site

**Fig. 1:**
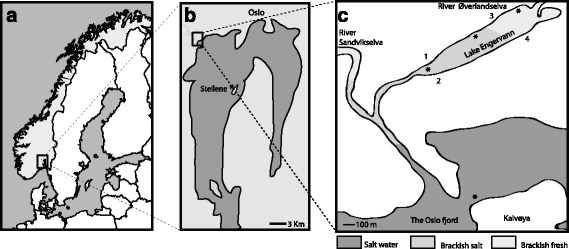
Geographical location of the brackish water Lake Engervann; **a**) location in Norway, **b**) location within the marine Oslofjord Fjord, and **c**) location within the Sandvikselva River drainage. In **c**) the upper (* close to the River Øverlandselva; freshwater influenced) and the lower (* close to the outlet river; saltwater influenced) sampling sites are given. Symbol * denotes also sampling stations of stable isotopes from putative prey items. Number 1–4 refers to the two replicate sampling sites for a benthic invertebrate survey by Halvorsen et al. [33] (data modified by us and presented in Additional file 8: Table S5). Salinity is illustrated as colors; dark grey = salt water, light grey = brackish-salt water and light grey = brackish-freshwater

Lake Engervann is a small (0.14 km^2^), shallow (max depth 3 m) brackish water lake situated in the lower reaches of the River Øverlandselva in the south-eastern part of Norway (59^o^53´46´´N, 10^o^31´56″E, Fig. 1). The main fish community comprises ninespine stickleback (*Pungitus pungitus*), threespine stickleback and the common goby (*Pomatoschistus microps*), with Atlantic salmon (*Salmo salar*), brown trout (*Salmo trutta*), and river lamprey (*Lampetra fluviatilis*) spawning in the inlet river. Less frequent are the black goby (*Gobius niger*), the sand goby (*Pomatoschistus minutus*), flounder (*Platichtys flesus*), European plaice (*Pleuronectes platessa*), cod (*Gadus morhua*), and eel (*Anguilla anguilla*). The lake is frequently visited by fish eating birds [32].

The upper section of Lake Engervann receives more fresh water than the lower section due to the inflowing fresh water from River Øverlandselva, while the lower section receives more salt water due to tidal influence (Fig. 1). There is large spatial and temporal variation in salinity, with conductivity varying between 60 and 3000 mS/m between sites and days in Lake Engervann reflecting the incoming fresh water from River Øverlandselva and incoming tide near the outlet of the lake (see [33]). In general, freshwater conductivity is rarely higher than 20 mS/m and seawater conductivity is commonly ca 5200 mS/m [34, 35]. In such, Lake Engervann can be evaluated as a typical brackish water area in Norway.

### Stickleback sample collection

Threespine stickleback were sampled using clear acrylic traps [36] in both the upper and lower sections of Lake Engervann during Autumn 2006 and Spring 2007 (Fig. 1, Table 1). We included the spatio-temporal comparison to test for differential patterns in ecology. All fish were euthanized using an overdose of MS222 and stored in 70% EtOH. A fishing permit (Fisketillatelse 10/2016; 2006/-SNO-1/TW) was granted by the Norwegian Directorate for Nature Management. Special care was taken to minimize suffering of fish. All sticklebacks were sorted into completely-, partially- and low plated morphs. The total number of individuals sampled and the total number of individuals within morphs were not recorded, but an unpublished survey based on a sample of 1000 sticklebacks throughout Lake Engervann showed a highly skewed morph frequency; 85% completely plated morphs, 13% partially plated morphs and 2% lowplated morphs. Therefore, to obtain an equal number of fish in each of the three lateral plate morphs in the two lake regions, and two time periods, we selected 14–25 fish within each of the 12 sampling groups (Table 1). Samples were used to test for differences in lateral plate number, ecology, parasites and genetic structure between the upper and lower lake region. Only sticklebacks from Spring 2007 was used for genetic structure analyses (Table 1).

**Table 1:** Body length measures (minimum-mean-maximum) in the 12 sample groups (sex pooled) of the three lateral plate threespine stickleback morphs in Lake Engervann based on sampling locations and dates

Lateral plate morph	Section	Sampling date	Body length (cm) min - mean - max	N males	N females	N fish for *Stn382*/μsat
*Completely plated (CPM)*	Upper part	Oct/Nov-2006	4.60–5.04 – 5.45	7	7	14/−
N fish total = 76:	Lower part	Oct/Nov-2006	4.55–5.08 – 5.70	7	8	15/−
N males = 38	Upper part	Mar/Apr-2007	4.70–5.22 – 5.70	12	10	22/22
N females = 38	Lower part	Mar/Apr-2007	4.75–5.27 – 6.75	12	13	25/25
*Partially plated (PPM)*	Upper part	Oct/Nov-2006	3.90–4.58 – 5.30	10	11	21/−
N fish total = 84:	Lower part	Oct/Nov-2006	4.15–4.77 – 5.35	10	4	14/−
N males = 42	Upper part	Mar/Apr-2007	4.25–4.84 – 6.45	9	15	24/24
N females = 42	Lower part	Mar/Apr-2007	4.50–4.99 – 5.90	13	12	25/25
*Low plated (LPM)*	Upper part	Oct/Nov-2006	3.35–4.24 – 5.50	9	10	18/−
N fish total = 76:	Lower part	Oct/Nov-2006	3.45–4.21 – 5.35	15	9	24/−
N males = 36	Upper part	Mar/Apr-2007	2.90–3.72 – 4.80	5	13	18/17
N females = 40	Lower part	Mar/Apr-2007	3.50–4.07 – 4.90	7	8	15/5

The number of fish analysed for morphology are reported for each sex, for *Stn382,* and microsatellites evaluated to be neutral in the last column

### Morphology

**Fig. 2:**
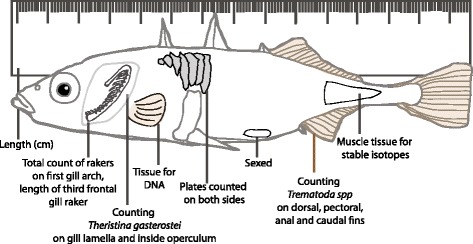
Graphic overview of measurements and counts performed on all sticklebacks

Body length from the tip of the nose to the end of the tail was measured, and the fish was sexed by visual inspection of gonads (Fig. 2). Lateral plates were counted on each side of the body, using a microscope, and summed for further analysis. All the sticklebacks were larger than the size when lateral plate development is assumed to have completed (estimated to be 27 mm in the completely plated morph in wild sticklebacks in the Oder and Vistula Rivers, Poland, by Banbura [37]). Two important traits often found relevant for tropic utilization in fish species were measured; the number of frontal gill rakers on the first right gill arch, and the length of the third frontal gill raker on the same gill arch using a microscope at 10 x magnification.

### Stable isotope analyses

Long-term diet and niche preferences, as a measure of the niche occupation were estimated using carbon (δ^13^C) and nitrogen (δ^15^N) stable isotope ratios in individual sticklebacks. Although data are not available for nitrogen isotope turnover in threespine sticklebacks, Grey [38] showed that δ^13^C in threespine stickleback muscle generally reflect a dietary history of approximately six months, a time span comparable to other north temperate fish species [39]. Stable isotopes reflect what the consumer has assimilated through its diet consumption in different habitats [40–42]. Here, δ^13^C reveal information on prey use along the saltwater-freshwater resource axis (depleted δ^13^C values reflect utilization of freshwater localized prey [41]. In contrast, δ^15^N values reveal information on trophic levels [40].

For analysis, a piece of the tail muscle was dissected out, dried for 24 h at 60 °C, ground, weighed, encapsulated in tin cups and analyzed in a stable isotope ratio mass spectrometer after methods in Harrod et al. [41]. Since C:N ratios differed among the three lateral plate morphs (ANOVA: F_2, 235_ = 41.85, *P* < 0.001), muscle lipid content also likely varies. Therefore all δ^13^C data was arithmetically lipid-normalized before further analysis [43].

Potential prey items were sampled using a dipnet and a sieve in the upper, middle and lower sections of Lake Engervann (Fig. 1). In a more marine influenced environment, benthic invertebrates along the shoreline of Kalvøya Island were sampled with a dip net and the pelagic habitat nearby the Steilene Island was sampled with a plankton net. Prey items were analyzed for stable isotopes. For each prey item, five or more specimens were pooled precluding a quantification of the variation within the species.

The putative stickleback prey items were; *Asellus aquaticus*, *Gammarus duebeni*, *Copepoda* spp., *Chironomidae* spp., *Palaemon adspersus, Neomysis integer, Pandalina profunda*, *Nudibranchia* spp., *Polychaeta* spp., *Planorboidea* spp., “unidentified snail spp.”, *Mytilus edulis*, *Balanus balanoides*, and *Chaegnata* spp*.* No zooplankton species were found in Lake Engervann despite several sampling efforts. The stable isotopic values for putative prey items are given in Additional file 1: Table S1.

### Parasites as ecological markers

Two ecto-parasites were additionally used as ecological markers to reflect long-term habitat- and diet, reflecting niche occupation. The choice of parasite species to be analyzed was based on their easy visual recognition and also their robustness with regard to sticklebacks being removed from storage in EtOH. Thus, we did not analyse *Gyrodactylus* spp. as the fish had been dried several times. First, the number of the crustacean copepod *Thersitina gasterostei* (family Ergasilidae) [Pagenstecher, 1861] was counted on the inner side of the operculum and on the muscle tissue close to the branchial arch on both sides of the fish. The sum of the copepods on both sides of the fish was used in analyses. *T. gasterostei* is a parasite on Holarctic euryhaline fishes [44], often on gasterosteids [45–47], and particularly on threespine stickleback [48].

The second parasite was an unidentified metacercaria of *Trematoda* spp. encysted only on the pectoral, dorsal, anal and caudal fins. The number of *Trematoda* spp. metacercaria cysts were counted on both pectoral fins, the dorsal, anal and caudal fins and then summed. To clarify the identity we used three primer-pairs to amplify a 1410 bp region (combined into one sequence) of the rDNA ITS gene [49] partly covering the ribosomal gene clusters 18S, ITS1, 5.8S, ITS2, 28S. PCR was performed using PuReTaq ready-to-go PCR beads (GE Healthcare), 1 mMol^− 1^ of each primer, and 5 ml of the extracted DNA in a 50-ml reaction volume. The thermal cycling was: 94 °C for 3 min; 35 cycles of 94 °C for 30 s, 55 °C for 47 s, and 72 °C for 1 min; and a final extension at 72 °C for 5 min. PCR products were purified by 10× diluted exoSAP-IT (USB). Cycle sequencing, using the same primers as in the PCR, was performed in 10-ml reactions using 2-ml BigDye terminator cycle sequencing ready kit (Applied Biosystems), 2 ml 5× sequencing buffer, 10 pmol primer, and 3-ml cleaned PCR product. One cyst from each of eight sticklebacks was sequenced. Data analyses were done in Sequencher 5.0 (Gene Codes Corporation, Ann Arbor, Michigan, USA) and aligned with the Crustal W algorithm in MEGA 6.0 [50] using default settings. Sequences were compared in BLAST (blast.ncbi.nlm.nih.gov/Blast.cgi) using a reduced set of 871 bp to increase number of comparisons to four sequences. Evolutionary analyses were conducted in MEGA6 with evolutionary history inferred using the Minimum Evolution (ME) method [51] with 1000 bootstraps [52]. Evolutionary distances were computed using the Maximum Composite Likelihood method [53] and are in units of number of base substitutions per site. The ME tree was searched using the Close-Neighbor-Interchange (CNI) algorithm [54] at a search level of 1. The Neighbor-joining algorithm [55] was used to generate the initial tree. Codon positions included were 1st + 2nd + 3rd + Noncoding. All positions with gaps and missing data were eliminated.

### Population genetic analysis

DNA was extracted from pectoral fins using standard proteinase K phenol chloroform protocol [56]. A set of 25 microsatellites, where 12 loci were a priori suggested to be neutral and 13 loci to be QTLs, were analyzed. Specific information about all the primers, PRC run conditions, binning and applied laboratory methods are reported in Le Rouzic et al. [13] and Klepaker et al. [57]. Two of the markers, *Stn381 and Stn382*, are situated in two introns of the Ectodysplasin gene (*Eda*) that is a major determinant of lateral plate morphs partitioning the completely plated, partially plated and the low plated morph [5, 13]. We analyzed individuals in the upper and lower part of Lake Engervann in spring 2007 (Table 1) as this should be sufficient for testing for potential population genetic structure. Genetic analyses were performed on all genotyped stickleback in Lake Engervann together assuming that all plate morphs belonged to a single population.

Microsatellites were first screened in MICRO-CHECKER 2.2.3 [58] to evaluate presence of stutter, allelic drop-out, homozygote excess and null-alleles. Four loci (*Stn152*, *Stn180*, *Stn211*, *Stn271*) showed homozygote excess/null alleles in two or three comparisons and were removed from all the further analyses.

To test if microsatellites were neutral or candidates for either directional or balancing selection, all 21 loci were run in LOSITAN [59, 60] under the stepwise mutation model (SMM) and the infinite alleles model (IAM). Here, we used 100,000 simulation with the “*Neutral mean Fst*” and “*Force mean Fst*” options when analyzing data separately for IAM and SMM models. For all the simulations, the two microsatellites *Stn381* and *Stn382* emerged as candidates for directional selection and was thus removed before analyzing the rest of the markers collectively. None of the remaining 19 microsatellites showed any signals of selection and were therefore inferred and used as neutral markers in population genetic structure analysis.

Genotypic linkage disequilibrium and deviations from Hardy-Weinberg equilibrium (HWE) were analyzed by the exact (probability) test estimated in GENEPOP 4.0.10 [61]. The results showed that a total of 4 loci differed from HWE, and only 3 after Bonferroni corrections. To be conservative, we thus removed the three loci (*Stn*178, *Stn*180 and *Gac*2111) from further population genetic analysis.

To test for population genetic structure, we used Bayesian population clustering in STRUCTURE 2.3.3. [62] with the 17 neutral microsatellites. We used an admixture model, correlated gene frequencies, 500,000 burn-in steps and 700,000 MCMC iterations, with K set to vary between 1 and 3 clusters with 5 replicates for each K. In addition, we conducted similar STRUCTURE analyses with the same burn-in steps and MCMC iterations, but now using the *locprior* option comparing (i) upper versus lower lake section regardless of morph (K:1–3, 5 replicates), (ii) three plate morphs (K:1–5, 5 replicates), (iii) or contrasting the morphs x location (K:1–6), 5 replicates. These additional analyses were performed with all loci (including loci deviating from HWE), but excluding *Stn381* and *Stn382* (being *Eda* linked loci), and null allele loci. The results were evaluated based on Evanno et al. [63] and Pritchard et al. [62] also using STRUCTURE-HARVESTER v0.6.93 [64]. We also ran a DAPC analysis (a principal component analyses) using *adegent* (http://adegenet.r-forge.r-project.org/).

### Statistical analysis

Differences in body length was tested using an ANOVA with the three lateral plate morphs and the two sampling dates (autumn 2006 or spring 2007) as predictor variables, grouping the upper and lower sampling sites in the lake together to increase statistical power. A post hoc Tukey HSD test was here used to test if lateral plate morphs differed significantly from each other in body length in the two time periods.

Using an ANCOVA we quantified the differences in lateral plate number among morphs while correcting for body length. The aim here was not to test if morphs were different, as we already have grouped them based on coverage of lateral plates – but rather to quantify the number of lateral plates in the three morphs. Here, we used the summed number of plates on both sides grouping all samples together in each morph.

As the two loci *Stn381* and *Stn382* provided similar information, given that they are both indel markers of *Eda*, we focused only on *Stn382* in subsequent analyses. This marker consistently gave two alleles and three genotypes in contrast to *Stn381* which may have three alleles and more genotypes [13, 17]. Association between *Stn382* genotypes *AA*, *Aa* and *aa* and the lateral plate morph categories (complete, partial and low plated) were tested using a contingency analysis. Due to the observed strong association between plate morph categories and *Stn382*-genotypes (see below) we only used plate morph category in further analyses.

We used general linear models to test if the number of frontal gill rakers and the 3rd gill-raker length on the first right lower gill arch were associated with the three lateral plate morphs (complete, partial or low plated), sex (male or female), or body length.

Using general linear models we tested if stable isotopic signatures of nitrogen (δ^15^N) and carbon (δ^13^C) were associated with sampling area (upper or lower part), sampling date (Autumn 2006 or Spring 2007), sex (male or female), lateral plate morphs (complete, partial or low plated) and body length. To evaluate if lateral plate morphs had different range in diet items (based on values of δ^15^N and δ^13^C) we tested if stable isotope variance differed among the three lateral plate morphs using the Levene’s test on the residuals from a correlation of body length vs stable isotopes.

Further, using parasite taxa as markers to reflect the ecological niche of lateral plate morphs, we estimated their *prevalence* (i.e. % of individuals in the population being infected) and *intensity* (i.e. mean value of fish being infected) in the whole Lake Engervann population, as well as separately for each of the three lateral plate morphs.

We tested variation in parasite infection (each taxon separately) using a generalized linear model with a Poisson distribution and log link, with the following predictors; sampling area (upper or lower part), sampling date (autumn 2006 or spring 2007), sex (male or female), lateral plate morphs (complete, partial or low plated), trophic traits (gill-raker number, 3rd gill-raker length), isotopes (δ^15^N, δ^13^C) and total body length.

All the statistical analyses were performed using the software JMP 9.0 [65].

## Results

### Genetic structure

All the four performed STRUCTURE analyses and the additional DAPC analysis suggested that the lateral plate morphs of threespine stickleback in Lake Engervann belonged to one single gene pool (K = 1). The result is reported in the Additional file 2: Table S2 and Additional file 3: Figure S3, while the data for genetic markers is given in Additional file 4: Table S3 and Additional file 5: Table S4.

### Body length

**Fig. 3:**
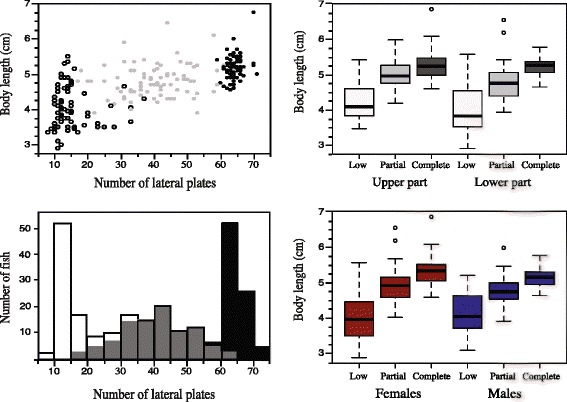
Association between number of lateral plates and body length (upper left panel), number of lateral plates and frequency (lower left panel), lateral-plate-morph specific body length in the upper and lower part of the lake (upper rigth panel), and lateral-plate-morph specific body length for males and females (lower rigth panel) in the three Lake Engervann sticklebacks. Black color denote the complete-, grey color denote partial-, and white color denote the lowplated morph

Body length differed significantly (whole model; R^2^ = 0.48, *N* = 236, d.f. = 235, *P* < 0.001) among the three lateral plate morph categories (effect test; F = 103.04, P < 0.001), but not between the two sampling seasons (F = 0.34, *P* = 0.562). The post hoc Tukey HSD test (q* = 2.36, N = 236, *P* < 0.05) on body length showed that all the three plate morphs differed significantly with the completely plated morph being largest (mean ± SD; 5.18 ± 0.34), the partially plated morph intermediate in size (4.81 ± 0.45), and the low plated morph the smallest (4.07 ± 0.60). Relationship between lateral plate number and body length, morphs and body length, and frequency classes are given in Fig. 3.

### Lateral plate morphs and *Eda* marker *Stn382*

The total number of lateral plates on both sides of the fish summed ranged between 8 and 71 (Fig. 3). Here, the completely plated morph had a mean (± SD) number of lateral plates of 64.2 ± 2.1, the partially plated morph a mean of 40.3 ± 10.3, and the lowplated morph a mean of 14.9 ± 5.9.

We observed a one-locus two alleles *Stn382- Eda* linked marker system with three genotypes (*AA: 218–218 basepair (bp) genotype*, *Aa: 158–218 bp genotype* and *aa: 158–158 bp genotype*) in all the Lake Engervann sticklebacks. The contingency analysis of *Stn382* vs lateral plate morphs revealed a significant association (χ^2^ = 230.37, *P* < 0.0001, *N* = 236). The highest percentage of *Stn382*-genotypes in morphs was; *AA* (89.5%) in the completely plated morph, *Aa* (59.5%) in the partially plated morph, and *aa* (73.7%) in the lowplated morph (all values are presented in Table 2).

**Table 2:** Associations among the three lateral plate stickleback morphs in Lake Engervann and the *Stn382* - *Ectodysplasin* genotypes. - denotes lack of observations of the specified *Stn382* - *Ectodysplasin* genotype in the complete- and lowplated morphs, respectively

Lateral plate morph	Ectodysplasin genotypes *Stn382*	N fish *Stn382*	% occurrence within morph	Plate number min-max	Plate number mean values	Plate number standard deviation
*Completely plated (CPM)*	*AA*	68	89.5	60–71	64.4	2.10
N fish total = 76	*Aa*	7	9.2	59–65	63.0	2.00
	*aa*	1	1.3	62–62	62.0	–
*Partially plated (PPM)*	*AA*	31	36.9	22–59	41.9	9.15
N fish total = 84	*Aa*	50	59.5	17–64	39.6	11.08
	*aa*	3	3.6	30–40	33.7	5.51
*Low plated (LPM)*	*AA*	0	–	–	–	–
N fish total = 76	*Aa*	20	26.3	10–33	16.1	6.64
	*aa*	56	73.7	8–37	14.4	5.64

### Trophic traits

The number of gill rakers on the first gill arch did not differ among morphs or varied with sex and size (Table 3). However, the length of 3rd gill-raker on the first right lower gill arch was significantly longer in males than females (Table 3).

**Table 3:** General linear mixed models on frontal gill raker counts on first right gill arc and on the 3rd gill-raker length on first right lower gill arch with predictor variables in the Lake Engervann threespine sticklebacks. - denote non-reported values

Test	Parameter	Estimate ± se	*F*	*P*
Number of frontal gill rakers on the first right gill arch	Intercept	20.637 ± 0.978	–	< 0.001
R^2^ = 0.02	Lateral plate morph - CPM*	0.319 ± 0.172	2.02*	0.135*
N = 236	Lateral plate morph - LPM*	−0.353 ± 0.189	–	–
*P* = 0.324	Sex	− 0.014 ± 0.098	0.02	0.888
Df = 4	Body length	−0.167 ± 0.208	0.65	0.423
3rd gill-raker length on the first right lower gill arch	Intercept	−0.029 ± 0.082	–	0.727
R^2^ = 0.656	Lateral plate morph - CPM#	0.011 ± 0.014	0.31#	0.736#
N = 236	Lateral plate morph - LPM#	− 0.010 ± 0.016	–	–
P < 0.001	Sex	−0.056 ± 0.008	46.86	< 0.001
Df = 4	Body length	0.243 ± 0.017	195.07	< 0.001

for * and # the presented F-values and *P*-values are only reported jointly for the three classes of the lateral plate morphs (*CPM* Completely plated, *PPM* Partially plated, *LPM* Lowplated) while parameter values reported separately for CPM and LPM

### Description of stable isotope values in putative prey items and sticklebacks

**Fig. 4:**
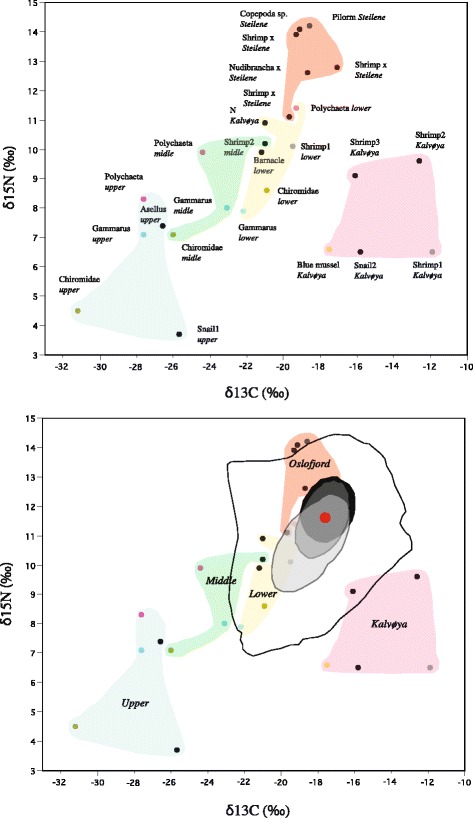
Stable isotope values from the three Lake Engervann stickleback lateral plate morphs and putative prey items based on δ^13^C and δ^15^N. The upper figure shows stable isotope values of different prey items sampled in different parts of Lake Engervann (upper, midle and lower sections) and outside in a more marine environment close to the Kalvøya Island and in a marine pelagic environment close to the Steilene Island in the inner Oslofjord. All prey items within the five nominal environmental categories are grouped using colors. In the lower figure is given the total range of individual stable isotope values in the threespine stickleback in Lake Engervann (thick black line) when grouping sampling time and sampling site into one analysis. Also, 50% density plots are given for the completely plated morph (black), partially plated morph (grey), and lowplated morph (light grey). The filled red circle denotes the mean value for all threespine sticklebacks analyzed from Lake Engervann

The putative stickleback prey items *G. duebeni*, *Chironomidae* spp. and *Polychaeta* spp. sampled in the lower, middle and upper part of Lake Engervann showed a pattern of more depleted values of δ^13^C and δ^15^N in the upper freshwater influenced part of the lake than in the lower brackishwater part of the lake (Fig. 4). The values for δ^13^C and δ^15^N differed in and among taxa along the salinity gradient from Lake Engervann to the more salinity influenced Kalvøya Island area and Steilene Island area (Fig. 4).

The variation in stable isotope values of sticklebacks in Lake Engervann (all data combined) ranged from − 22.5 to − 13.0 ‰ for lipid-corrected δ^13^C and between 7.0 and 14.3 ‰ for δ^15^N (Fig. 4). The variance was 2.5 for δ^13^C and 1.7 for δ^15^N in all stickleback. This minimum-maximum range in stable isotope values of sticklebacks is visualized by the thin black line in the convex hull plot of their putative prey items in Fig. 4. The mean values of δ^13^C and δ^15^N were similar in morphs. Mean δ^13^C values (mean ± SD) for each morph were; completely plated: − 17.1 ± 1.3, partially plated: − 17.1 ± 1.3 and the low plated morph: − 17.8 ± 1.9. Mean δ^15^N values were more variable; completely plated: 12.0 ± 1.0, partially plated: 11.6 ± 1.2 and the low plated morph: 10.9 ± 1.5.

### Predictors of stable isotope values in sticklebacks

Variation in δ^15^N was significantly associated with sampling area, having larger values in the lower part of the lake, with larger values of δ^15^N in autumn 2006 than in spring 2007, and being correlated with body length as larger fish had higher values of δ^15^N (Table 4). The variation in δ^13^C generally followed the same pattern, but having more ^13^C–enriched values in the upper part than in the lower part of Lake Engervann (Table 4).

**Table 4:** General linear mixed models on niche/diet preferences by the use of stable isotopic responses of δ^15^N and δ^13^C with predictor variables for the threespined stickleback in Lake Engervann. - denote non-reported values

Test	Parameter	Estimate ± se	Df	*F*	*P*
Stable isotopic values of nitrogen (δ^15^N)	Intercept	9.380 ± 0.772	–	–	< 0.001
R^2^ = 0.21	Sampling area	0.238 ± 0.077	1	9.50	0.002
N = 236	Sampling date	−0.210 ± 0.078	1	7.31	0.007
P < 0.001	Sex	−0.086 ± 0.077	1	1.25	0.265
Df = 6	Lateral plate morph - CPM*	0.258 ± 0.135	1	2.37*	0.095*
	Lateral plate morph - LPM*	−0.313 ± 0.149	1	–	–
	Body length	0.470 ± 0.164	1	8.21	0.005
Stable isotopic values of carbon (δ^13^C)	Intercept	−20.86 ± 0.943	–	–	< 0.001
R^2^ = 0.20	Sampling area	0.193 ± 0.095	1	4.16	0.043
N = 236	Sampling date	−0.474 ± 0.095	1	24.97	< 0.001
P < 0.001	Sex	0.001 ± 0.094	1	0.00	0.988
Df = 6	Lateral plate morph - CPM#	−0.117 ± 0.165	1	1.45#	0.237#
	Lateral plate morph - LPM#	− 0.105 ± 0.182	1	–	–
	Body length	0.762 ± 0.201	1	14.45	< 0.001

for * and # the presented F-values and *P*-values are only reported jointly for the three classes of the lateral plate morphs (*CPM* Completely plated, *PPM* Partially plated, *LPM* Lowplated) while parameter values reported separately for CPM and LPM

A Levene test (F = 4.84, *N* = 236, df = 233, 2, *P* = 0.009) showed that the variance of δ^13^C was larger in the low plated morph (variance: 3.15, *N* = 76) than in the partially plated (1.77, *N* = 84) and the completely plated morph (1.89, N = 76). The same pattern was seen for δ^15^N (F = 9.40, N = 236, df = 233, 2, *P* < 0.001), where the low plated morph (variance: 2.11, N = 76) had a significantly larger variance than the partially plated (1.27, N = 84) and the completely plated morph (1.01, N = 76). The partially plated morph and completely plated morph did not differ in δ^13^C and δ^15^N variance.

### Parasite infection in sticklebacks

#### T. Gasterostei

**Fig. 5:**
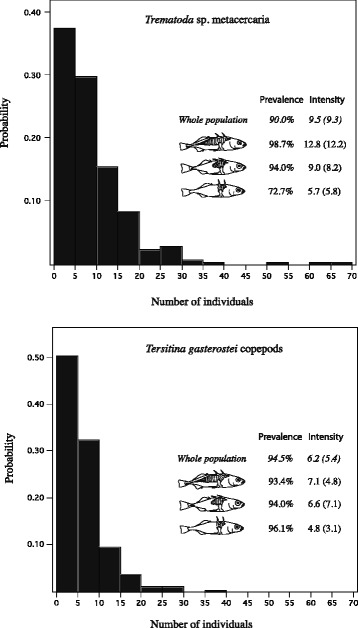
Probability of frequency classes of *Trematoda* spp. cysts summed over the caudal, anal, dorsal, and both pectoral fins (upper figure), and probability of frequency classes of the crustacean copepod *Theristina gasterostei* on the inner side of operculum and gills (lower figure) found on the three Lake Engervann stickleback lateral plate morphs. Bars denote the whole population of sticklebacks in Lake Engervann with additional information on prevalence and intensity (standard deviation) for whole population and for the complete-, partial- and lowplated morphs

The total number of *T. gasterostei* on individual sticklebacks in the whole Lake Engervann stickleback population (combining plate morphs, time and locations) approached a Poisson distribution (Fig. 5 lower panel) (see also Additional file 6: Figure S1. The number of *T. gasterostei* ranged between 0 and 37 (mean ± SD, 5.8 ± 5.4) (Fig. 3), with only 13 of 236 sticklebacks lacking *T. gasterostei* giving a prevalence of 94.5% and mean infection intensity (i.e. only infected individuals) of 6.2 (± 5.4).

The prevalence and mean intensity of infection was quite similar among the morphs. In the completely plated morph (combining time and locations) 5 out 76 individuals lacked *T. gasterostei* giving a prevalence of 93.4% and mean intensity of 7.1 (±4.8). For the partially plated morph (combining time and locations), 5 of 84 individuals lacked *T. gasterostei* giving a prevalence of 92.9% and a mean intensity of 6.6 (±7.1). In the lowplated morph (combining time and locations), 2 out of 71 individuals lacked *T. gasterostei* thus giving a prevalence of 97.2% and a mean intensity of 4.8 (± 3.1).

The number of *T. gasterostei* was significantly associated with several predictor variables (Table 5). Here, sticklebacks sampled in the upper part of the lake had more *T. gasterostei* than in the lower part. Females had more *T. gasterostei* than males. Sticklebacks with fewer and shorter gill rakers had more *T. gasterostei* than sticklebacks with more and longer gill rakers. Furthermore, sticklebacks with higher δ^15^N values had more *T. gasterostei* than those with lower values. Sticklebacks with larger body length had more *T. gasterostei* than fish with smaller body size (Table 5).

**Table 5:** General linear mixed models of counts of the parasitic copepod *T. Gasterostei* and counts of *Trematoda* spp*.* cysts with predictor variables for the threespined stickleback in Lake Engervann. - denote non-reported values

Test	Parameter	Estimate ± se	Df	χ2	*P*
Number of *T. gasterostei* (Poisson distribution, log link)	Intercept	3.524 ± 0.724	–	–	< 0.001
χ^2^ = 231.92	Sampling area	−0.078 ± 0.028	1	7.80	0.005
N = 236	Sampling date	0.055 ± 0.030	1	3.30	0.069
*P* < 0.001	Sex	0.072 ± 0.031	1	5.27	0.022
Df = 10	Lateral plate morph - CPM*	0.030 ± 0.046	1	0.46*	0.796*
	Lateral plate morph - LPM*	−0.032 ± 0.053	1	–	–
	Gill raker number	−0.073 ± 0.019	1	15.01	< 0.001
	3rd gill-raker length	−0.676 ± 0.220	1	9.54	0.002
	δ^15^N	−0.193 ± 0.024	1	67.69	< 0.001
	δ^13^C	0.033 ± 0.021	1	2.52	0.112
	Body length	0.668 ± 0.082	1	64.78	< 0.001
Number of *Trematoda* spp*.* (Poisson distribution, log link)	Intercept	−0.458 ± 0.583	–	–	0.431
χ^2^ = 784.95	Sampling area	0.114 ± 0.024	1	23.12	< 0.001
N = 236	Sampling date	−0.116 ± 0.025	1	20.99	< 0.001
P < 0.001	Sex	−0.063 ± 0.026	1	5.913	0.015
Df = 10	Lateral plate morph - CPM#	0.214 ± 0.037	1	39.25#	< 0.001#
	Lateral plate morph - LPM#	−0.266 ± 0.048	1	–	–
	Gill raker number	0.009 ± 0.015	1	0.410	0.524
	3rd gill-raker length	0.566 ± 0.173	1	10.68	0.001
	δ^15^N	0.190 ± 0.026	1	55.58	< 0.001
	δ^13^C	0.137 ± 0.018	1	56.81	< 0.001
	Body length	0.365 ± 0.073	1	24.67	< 0.001

for * and # the presented χ2-values and *P*-values are only reported jointly for the three classes of the lateral plate morphs (*CPM* Completely plated, *PPM* Partially plated, *LPM* Lowplated) while parameter values reported separately for CPM and LPM

#### *Trematoda* spp.

All the eight sequenced cysts collected from the sticklebacks were identical (Genbank accession number KY620038).The sequence clustered most closely with the heterophyid trematode causing the black-spot disease *Cryptocotyle lingua* (Genbank Accession Number: KJ641524 [66] with 956 of 1006 bp matched (97% query coverage, identification 100%). The next best hit was *Cryptocotyle lingua* (Genbank Accession Number: KJ641518; [66]) with 950 of 1006 bp matched (96% query coverage). As our sequence had a bootstrap value of 100% grouping out from *C. lingua* (and the outgroup of *Pygidiopsis genata* (AY245710 [67], our metacercaria was evaluated as being significantly different from *C. lingua* (Additional file 7: Figure S2). The taxonomy is currently unknown.

The summed distribution of *Trematoda* spp. on the pectoral, pelvic, dorsal, anal and caudal fins (combining plate morphs, time and locations) approached a Poisson distribution (Fig. 5 upper panel) (see also Additional file 6: Figure S1. The number of *Trematoda* spp. ranged from 0 to 65 (mean ± SD; 8.42 ± 9.25) (Fig. 3), where 29 of 236 fish lacked *Trematoda* spp. giving a prevalence of 89.9% and a mean intensity of 3.5 (±1.4).

Prevalence and mean infection intensity varied among morphs*.* In the completely plated morph (combining time and locations) only 1 of 76 fish lacked *Trematoda* spp. giving a prevalence of 98.7% and a mean intensity of 12.8 (±11.2). For the partially plated morph, 5 of 84 fish lacked *Trematoda* spp. giving a prevalence of 94.0% and a mean intensity of 12.8 (±8.2), while in the lowplated morph 22 out 71 fish lacked *Trematoda* spp. giving a prevalence of 69.0% with a mean intensity of 5.4 (±5.0).

The number of *Trematoda* spp. was significantly associated with a set of variables (Table 5). Here, sticklebacks in the lower part of the lake had more *Trematoda* than in the upper part. More *Trematoda* spp. were recorded in Autumn 2006 than in Spring 2007. Males had more *Trematoda* spp. than females. The lowplated morph had fewer *Trematoda* spp. than the other two morphs. Fish with larger gill raker length had more *Trematoda* sp. than fish with smaller raker length. Furthermore, stickleback with larger values of both δ^15^N and δ^13^C had more *Trematoda*. spp. cysts. Finally, fish with larger body length had more *Trematoda* spp. cysts than smaller sticklebacks (Table 5).

## Discussion

### One panmictic gene pool comprising all the three sympatric lateral plate morphs

The three morphs of threespine stickleback in Lake Engervann belonged to one single population revealed as one genepool. Conversely, we observed marked between morph differences in body length and lateral plate counts indicating limited gene flow (or divergent selection) among morphs for such traits. As such, it may still be that the underlying genes for heritable traits have a lower degree of gene flow among plate morphs if associated with divergent adaptive traits, behaviour, niche preferences or life history. Such heterogeneous genomic differentiation was observed in Baltic Sea sticklebacks by DeFaveri et al. [68] and Guo et al. [30]. Based on the *Eda* results, it seems that the plate morphs in Lake Engervann are more or less genotypically distinct while yet sharing the same neutral genepool. This could suggest that morphs may differ in traits, behavior and life history due to associations with the *Eda* haplotype block, or alternatively that traits are free to be exposed to natural selection and evolve without genetic constraints if residing on different chromosomes. Natural selection may fine-tune important adaptive traits in the *EDA* haplotype block while still allowing for neutral genes to flow among the three lateral plate morphs.

### Associations among *Eda* genotypes, lateral plate counts and lateral plate morphs

The range in plate number observed in Lake Engervann sticklebacks (both sides summed; 8–71) covers most of the variation seen in populations of the threespine stickleback in the Holarctic [7, 69–71]. The three *Eda Stn382*-genotypes *AA*, *Aa* and *aa* were clearly associated with the three plate morphs where the *AA* genotype (89.5%) dominated in the completely plated morph, the *Aa* genotype (59.5%) in the partially plated morph and the *aa* genotype (73.7%) in the low plated morph. Similar patterns of *Eda* markers (*Stn380, Stn381*, *Stn382*), plate morphs and plate number have been found in other studies, although with variation in relative percentages of genotypes in morphs [5, 13, 16–18, 72, 73]. An interesting comparison is the threespine stickleback in Lough Furnace, Burrishole Catchment, Western Ireland where a small bodied freshwater stationary form enters brackish water (8.3–29.7 ppt) area living together with a larger brackish water resident form and an even larger anadromous form of stickleback [16]. The Irish plate morphs are comparable in lateral plate number and body size to the three plate morphs in Lake Engervann, where similarities are also evident in the association between lateral plate morphs and the *Eda* marker genotypes at *Stn382*. The repeated association between lateral plate morphs and salinity environments from ancestral marine to derived freshwater habitats suggests adaptive loss of lateral plates [2, 3, 6, 7]. In support, genetic studies in sticklebacks document that *Eda* is under divergent selection in contrasting environments [5, 14] where also other traits in the *Eda* haplotype block could be under linkage-disequilibrium-based hitchiking and pleiotropic selection. The proportion of plate morphs varies in the Holarctic, where similar patterns as seen in Lake Engervann are found in brackish water and young freshwater lakes along the whole Norwegian coast [3].

### Ecological niche diversity of plate morphs based on stable isotopes and parasites

The ecological niche of an individual comprises the temporally framed accumulated habitat use and food preferences, which can also change during ontogeny through niche shifts. We did not perform quantitative sampling aiming at estimating the density of putative prey species for sticklebacks, but we evaluated that the most available invertebrate species would be the most likely prey for threespine stickleback in Lake Engervann based on an earlier survey in Lake Engervann by Halvorsen et al. [33] (Additional file 8: Table S5). In that study, the most common species were *Chironomidae*, *Oligochatea* and *Gastropoda* (*Hydrobia ulvae*). We did not analyze stomach content in the sticklebacks in Lake Engervann, but other studies have found that a diversity of prey species are consumed by threespine stickleback in brackish water environments spanning benthic-littoral zone animals such as molluscs, ostracods, chironomida, cladocerans, ciliates, orthocladiinae, and copepods in the pelagic zone [74–79].

#### Stable isotope signatures

In general, the patterns in the stable isotope data suggest that sticklebacks were foraging in the middle-lower part of Lake Engervann and more marine environments, rather than in the upper lake area (Fig. 4). The total range in δ^13^C and δ^15^N values in the sticklebacks ranged − 22.5 to − 13.0 ‰ and 7.0 to 14.3 ‰, respectively. Based on one prey item, *Chironomidae* spp. δ^13^C and δ^15^N values showed large variation from upper-middle and lower parts of Lake Engervann from − 31.2 to − 26.0 to − 20.9 ‰ and 4.5 to 7.1 to 8.6 ‰, respectively (see other putative prey items in Additional file 1: Table S1). Despite the impression that the three lateral plate morphs in Lake Engervann had generally similar diets based on stable isotopes when visualized in the biplot of δ^13^C versus δ^15^N, we observed interesting statistical differences. First, we found that the low plated morph had larger variance in δ^15^N and δ^13^C than the two other morphs. This finding may suggest that the low plated morph is more explorative in its habitat use and prey preferences than individuals in the two other morphs. Secondly, there was a spatial component in niche use in Lake Engervann as sticklebacks in the lower lake section were more enriched for ^15^N and ^13^C than in the upper section. This pattern may reflect freshwater stationary feeding behavior on a simple food chain in the upper part of the lake and a migratory foraging behavior on a more complex food chain with higher diversity of prey at different trophic layers in the lower part of the lake - with foraging migrations into marine environments. Also, it may reflect a difference in the relative amount of benthic v pelagic food consumed in each of the zones. Alternatively, it may reflect different salinity-isotope influences in the prey items and stationary sticklebacks in the two areas in Lake Engervann. There was also a seasonal component showing that sticklebacks were more enriched in ^13^C and ^15^N in the Autumn than Spring, likely reflecting differences due to prey production in the two seasons and/or accumulated diet diversity increasing over time. In another study by Nordström et al. [80] studying threespine stickleback in the Bothnian Bay, Finland, threespine stickleback isotope mean values varied around − 21 for δ^13^C and 10 for δ^15^N in a brackish water salinity regime (i.e. 5.2–6.0). In a previous study of 25 North Norwegian threespine stickleback populations we found that δ^13^C ranged from − 30.5 to − 13.2 (mean ± SD; − 22.3 ± 3.6) and that δ^15^N ranged from 5.0 to 15.3 (8.9 ± 1.8) (baseline data used in Østbye et al. [81]). Ravinet et al. [82] studied two stickleback clades in Japan and found that the upstream populations had a mean δ^13^C of − 19.4 ‰ and δ^15^N of 11.9 ‰. Other populations closer to marine environments had means of − 15.8 to − 18.0 ‰ for δ^13^C and 13.9 to 12.8 ‰ for δ^15^N. The total mean range in that study was − 15.1 to − 18.0 for δ^13^C and 11.9 to 14.0 for δ^15^N, with individual variation of ca − 35.0 to − 15.0 for δ^13^C and 6 to 15 for δ^15^N. It was found that the Japan Sea clade were less adapted to fresh water having a higher trophic position utilizing more brackish - marine areas than the Pacific clade. The pattern and range in δ^13^C (− 22.5 to − 13.0) and δ^15^N (7.0 to 14.3) in Lake Engervann resembles these comparative studies implying that the lateral plate morphs in Lake Engervann utilize fresh, brackish and marine areas.

#### Prevalence and intensity of T. Gasterostei

It is not fully understood if *T. gasterostei* is stenohaline, preferring brackish water habitats or if it is euryhaline and able to handle a large range in salinity. A number of studies have described *T. gasterostei* from threespine stickleback and other stickleback species in the Holarctic [79, 83–96]. Based on these studies it appears that this parasite can be found in a salinity range of 0.5***–***32, but are mostly found in brackish water and rarely in fresh water. Thus, it seems reasonable to infer that this parasite is largely distributed and transmitted in brackish water. The prevalence and infection intensity in these studies suggested that infection levels were consistently higher in brackish water than in fresh water and marine environments.

The prevalence of *T. gasterostei* was similar and high for all three lateral plate morphs in Lake Engervann, with values of 93.4%, 94.0%, 96.1%, for complete, partial and low plated morphs, respectively. The mean intensity was highest in the complete morph (7.1), lower in the partial morph (6.6) and lowest in the low plated morph (4.8), with large standard deviations. Studies of Baltic Sea threespine sticklebacks close to Poland by Morozinska-Gogol [97–99] revealed a prevalence range of 7.3–100% and a mean intensity range of 2.9–113 of *T. gasterostei*. Prevalence differed among the three Baltic Sea stickleback lateral plate morphs with 69.6%, 67.6% and 44.7% reported for the complete, partial and low plated morphs, respectively. These values are different from our results which are lower in prevalence, but close to the mean intensity range in the Baltic Sea plate morphs (a mean intensity of 6.2 observed in Lake Engervann). Valdez [90] found in a study of Alaskan threespine stickleback that 41% of the low plated morph compared to 21% of the partially plated morph (few completely plated fish caught) were infected. In contrast, Walkey et al. [89] observed that *T. gasterostei* was most common in the marine form (completely plated morph) in England. In Canadian threespine stickleback, Peddle [94] found a prevalence of *T. gasterostei* of 8% in full salinity (32.1) seawater. A comparison of *T. gasterostei* among studies should consider that this species has a seasonal distribution as seen in Mecklenburg area in the Baltic Sea, where the intensity of *T. gasterostei* was higher in June and July [93]. We found a lower infection of *T. gasterostei* in Autumn than in Spring which may reflect a seasonal influence due to the life history of the parasite. However, in Poulin and FitzGerald [47] the highest infection of *T. gasterostei* was found in three stickleback species in September–November in a salt marsh in Quebec, Canada. Donoghue [91] report that the highest prevalence and intensity of *T. gasterostei* on ninespine stickleback during the year was in November and May–June with low prevalence in March. Based on these different studies, in different stickleback species, it is not easy to see a clear temporal pattern in infection-intensity dynamics. Our result in Lake Engervann seems to be within the range of values in other studies, but are somehow distinctive in their high prevalences and relatively low mean intensities.

In the statistical analyses we found that sticklebacks sampled in the upper part of the lake had more *T. gasterostei*, and that sticklebacks with higher δ^15^N values had more *T. gasterostei*. Further, larger-bodied sticklebacks had more *T. gasterostei* than smaller fish, and females had more *T. gasterostei* than males. Sticklebacks with fewer and shorter gill rakers were more infected with *T. gasterostei*. These findings may collectively imply divergent foraging modes and life history adaptations towards divergent habitats within Lake Engervann as well as towards marine habitats.

#### Prevalence and intensity of Trematoda spp.

With regard to our observed *Trematoda spp*., the sequence suggested a genetic relationship to the trematode *Cryptocotyle lingua* which is responsible for the “black spot disease”*.* However, in contrast to the black spot disease which only occur on the dermal surface on the body - our cysts were only encysted in the fins and less pigmented. Also, our *Trematoda* spp. cysts appear slightly larger than blackspot cysts from other localities in Norway (visual evaluation; no statistical analysis). Our *Trematoda* spp. could potentially be e.g. *Cryptocotyle concavum* as this species have been described from stickleback before [100] and this taxon has larger cysts than its sister taxon *C*. *lingua* [101]. In addition, the common goby appears regularly in Lake Engervann which is a reported second intermediate host after the first intermediate host being the snail *Hydrobia stagnalis* and the final host being *Larus ridibundis* for *C. concavum* (more species of gulls can be final hosts; see Zander et al. [102]). Unfortunately, *C. concavum* has no sequences entered in Genbank. It may be that our *Trematoda* spp. has a transmission route that resembles *Cryptocotyle lingua* which is being transmitted through the intermediate host periwinkles (*Littorina littorina*) and its main host *Larus* spp. [103] or resembles *C. concavum* transmitted via *H. stagnalis* snails [102] to its main host *Larus* spp. *Littorina littorina* is more marine than *Hydrobia ulvae*, the dominant gastropod in Lake Engervann. Thus, *Trematoda* spp. may be transmitted via second intermediate host of *H. ulvae* in Lake Engervann or that sticklebacks with high infection levels are foraging in more marine environments than in Lake Engervann*.* Support comes from Möller [103] who found in a salinity-temperature and survival experiment of different stages of *C. lingua* that a salinity below 4 resulted in 50% of eggs developing and that salinities above 8 increased the living time of free swimming cercaria. It was also shown that this parasite preferred lower temperatures that were associated with increased survival time. Lake Engervann is shallow, resulting in higher mean temperatures, as well as more marked temperature fluctuations, relative to more thermally-stable marine environments. As such, it seems likely that transmission of our *Trematoda* spp. occurs more in marine-like habitats.

We observed that sticklebacks in the lower area had more *Trematoda spp.*, and a higher infection of *Trematoda* spp. in Autumn than Spring, which fits with seasonal foraging migrations into the marine environment. We have observed that in the middle of summer often there is a very low catch of threespine stickleback in Lake Engervann which could support such a seasonal migration pattern. Furthermore, the finding that the low plated morph had fewer *Trematoda* spp. than the other morphs, and that sticklebacks with larger gill raker length had more *Trematoda* spp., may also support a seasonal migration scenario that may differ among morphs. This pattern may fit with theoretical expectations of a divergent trait-associated benthic-pelagic foraging mode assuming that *T. gasterostei* is transmitted in littoral-benthic areas in brackish water, while *Trematoda* spp. is transmitted in more marine influenced areas where sticklebacks may also forage more on pelagic zooplankton.

### Body length patterns in the stickleback lateral plate morphs in Lake Engervann

The three lateral plate morphs in Lake Engervann differed significantly in body length with the low plated morph being smallest and the completely plated morph being largest, with the partially plated morph in between. Ravinet et al. [16] found that the three plate morphs of the threespine stickleback that coexisted in the Lough Furnace, Burrishole Catchment, Western Ireland were genetically segregated. Here, a small bodied freshwater stationary form seemed to enter (or being washed in to the lake passively) brackish water areas where a larger brackish water resident form and an even larger anadromous form of stickleback occurs. In contrast, the three Lake Engervann plate morphs belonged to one genetic population. Furthermore, our low plated morph is more genetically related to the partial and complete morph in the lagoon than to an upstream low plated morph in the Sandvikselva River (unpublished results).

Threespine sticklebacks in Lake Engervann live in a brackish water environment, but we do not know if they are truly stationary in the lake or if they are conducting migrations into the sea or to fresh water. However, our stable isotopic data suggest sticklebacks use both brackish water and marine areas for feeding. In a common garden experiment, Marchinko & Schluter [20] observed that offspring of reduced lateral plate morphs (partial and low) grew equally well as the offspring of the completely plated morph if raised in salt water, but that the former group grew better in fresh water. We are aware of no laboratory experiment looking at specific growth patterns of the completely plated, partially plated and low plated morphs raised under brackish water conditions. In another set of laboratory rearing experiments of stationary and anadromous threespine sticklebacks in the Navarro River, USA, Snyder & Dingle [26] and Snyder [104] suggested a genetic basis for life history variation implying different migratory lifestyles had evolved as reflected in genetic variation for size and growth. We expect that the three lateral plate morphs in Lake Engervann should have attained the same body size if they lived in the same environment and if they had the same niche, when assuming no genetic influence on growth by the three *Eda* genotypes. An interesting study in that regard was conducted by Robertson et al. [23] who found the *aa* low plated fish had a lower growth rate than the faster growing *AA* completely plated fish in an experiment exposing three *Eda* genotypes to natural marine and freshwater sites, which could support our results. Bowles et al. [105] found a genetic basis for body size variation between anadromous and derived lacustrine populations of threespine sticklebacks in southwest Alaska when crossing and raising offspring in water of salinity 4–6. The authors concluded that body size is a heritable trait that could be plastic as well as influenced by natural- and sexual selection. Thus, our results suggest that different body lengths of the lateral plate morphs may reflect either phenotypic plasticity in divergent niches and/or that growth, foraging and life histories are contingent upon genetic architecture associated with the *Eda* block itself.

### Ongoing divergent niche differentiation with high gene flow in the lagoon?

The utilization of a diversity of habitats, and thus niches, could render individuals and populations exposed to natural selection in divergent salinity environments, where selection introduce and trade-off conflicting trait adaptations. In the brackish water zone, the threespine stickleback may consist of such diversity in phenotypes and physiological traits due to hybridization among fresh water, brackish water and marine sticklebacks. Thus, adapting to the brackish water zone itself may be difficult due to high gene flow for the sticklebacks that are prone to be more brackish water resident. In our study, we argue for finding signs of ongoing divergent niche adaptation among the three lateral plate morphs based on ecologically related measures such as stable isotope values and parasites. Further, the ecological preferences may somehow be associated with the plate morphs through the *Stn382* link to the *Eda* haplotype block since sticklebacks belonged to one panmictic gene pool. The higher stable isotope variance seen in the low plated fish in Lake Engervann may imply a more explorative behaviour - which may fit with the salinity preference experiment by Barrett et al. (2009b). The plate morphs in Lake Engervann also have different foraging efficiency on pelagic *Daphnia* and benthic *Chironomidae* [106]. In an experiment on standard metabolic rate (SMR), as a cost for osmoregulation in salinity environments, we found that sticklebacks were able to move among salinity environments without short-term metabolic costs, irrespective of environment of origin from marine, brackish or fresh water, with no differences in SMR found among the three lateral plate morphs in Lake Engervann [107]. This suggests that these three plate morphs may be equally successful in different salinity environments, at least on a short time scale. This physiological ability thus seems to have been evolutionarily conserved within each plate morph. Further support for a multi-adaptive-trait association with the *Eda* block can be found in Greenwood et al. [22], who documented that schooling behavior was associated with *Eda* in benthic low plated and pelagic completely plated morphs. Also, the study by Robertson et al. [23] adds another dimension to the adaptive diversification of *Eda* genotypes and plate morphs as genotypes were found to differ in immune system genes associated with parasite load. The presence of sticklebacks from marine to brackish and freshwater environments intuitively suggest highly divergent selection pressures where one should expect adaptations to evolve. An experimental study by Peeke & Morgan [27] found differences in the response to behavioural stimuli associated with aggression, courtship and feeding in sticklebacks in marine, estuarine and upstream freshwater river in California, USA. This points to adaptive behavioural modifications along the marine-freshwater transect. Barrett et al. [108] observed in an experiment that cold tolerance could evolve rapidly in threespine stickleback from marine to fresh water, which could be different in colder brackish water lagoons than in warmer marine environments. Interestingly, DeFaveri and Merilä [109], De Faveri et al. [68] and Guo et al. [30] observed signatures of adaptive genetic differentiation associated with salinity and temperature gradients in the brackish water Baltic Sea threespine stickleback. These authors suggested that the same adaptive processes could occur in brackish water as in the marine-freshwater environment transects. Thus, the more stationary threespine sticklebacks in Lake Engervann could experience more variable temperature selection pressures than the more migratory-marine individuals. Further, being stationary in brackish water may also prime a temperature selection pressure for cold tolerance that could be benign when colonizing fresh water. Jones et al. [6] found a repeated pattern documenting that three haplotype blocks were important in differentiating marine and freshwater sticklebacks on the Holarctic scale, comprising genes associated with mucus production, salinity tolerance and lateral plate plates. Moreover, De Faveri et al. [110] found that physiological adaptation in freshwater-marine stickleback populations can follow alternative routes for genomic adaptation. A set of studies show that both de novo evolution and selection on standing genetic variation are involved in the marine-brackish water-freshwater stickleback adaptation [5, 6, 68, 109, 110]. Further research should aim at investigate the broad-spectra of physiology, and its plasticity, in different plate morphs to reveal their relative contribution upon adaptive radiation potential in different environments.

## Conclusions

The association of morphs, *Eda*, body length and ecology suggest that the three lateral plate morphs in the brackish water Lake Engervann are experiencing ongoing divergent selection for niche and migratory life history strategies even under high gene flow level as all the three plate morphs belonged to the same gene pool. The brackish water zone may as such act as a generator in a continuous adaptive iterative process where the plate morphs/*Eda* genotypes derive adaptive traits, resulting in novel functional genomic diversity to be selected upon in the different environments.

## Additional files

Additional file 1: Table S1. Summary statistics of stable isotopic values of putative prey items for the threespine stickleback sampled in the brackish water Lake Engervann and marine areas. (PDF 176 kb)

Additional file 2: Table S2. STRUCTURE-HARVESTER results for the Lake Engervann sticklebacks. (PDF 640 kb)

Additional file 3: Figure S3. DAPC analyses (Dicriminant analysis of principal components) in *adegenet* (Jombart 2008) performed on the three lateral plate morphs of threespine stickleback from Lake Engervann. (PDF 299 kb)

Additional file 4: Table S3. Microsatellite raw data used for the Lake Engervann threespine stickleback. (XLSX 42 kb)

Additional file 5: Table S4. *Eda* locus *Stn382*, body length, sex and location for the Lake Engervann threespine stickleback. (XLSX 16 kb)

Additional file 6: Figure S1. The distribution of *Theristina gasterostei* on the left and rigth side of the gill-region and the distribution of *Trematoda* spp. cysts on the pectoral, dorsal, anal and caudal fin in the threespine stickleback in Lake Engervann, Norway. Dark bars denote males and ligth grey bars denote females. All the analysed fish sampled in Lake Engervann have been pooled (*N* = 236). Note different y-axes. (EPS 503 kb)

Additional file 7: Figure S2. Evolutionary relationships of four taxa compared using a 1006 bp long sequence of the rDNA ITS gene visualized in a minimum evolution tree. The two sequences of *Cryptocotyle lingua* are from Atlantic cod (*Gadus morhua*) while the unknown *Trematoda* spp. is from threespine stickleback in Lake Engervann. (EPS 408 kb)

Additional file 8: Table S5. The invertebrate fauna sampled on four locations (with an equal sampling effort performed on each locations) in the littoral area in the brackish water Lake Engervann on two dates (data report number of individuals sampled with the percentage contribution summed over two dates and combined for stations in the lower (Lower %) and upper sections (Upper %), and for the four sites and two dates combined in last column (Total %) (all data are taken from Halvorsen et al. [1]. Sampling sites 1 and 2 are from the lower and southern section on each side of the lake while site 3 and site 4 is situated on each side in upper northern part of the lake (see Fig. 1). (PDF 199 kb)

Additional file 9: Table S6. Other raw data on the Lake Engervann threeespine stickleback. (XLSX 71 kb)
