# Supplementary material for: Ongoing niche differentiation under high gene flow in a polymorphic brackish water threespine stickleback (Gasterosteus aculeatus) population
Source: BMC Evol Biol. 2018 Feb 5;18:14. doi: 10.1186/s12862-018-1128-y (PMC5800020; doi:10.1186/s12862-018-1128-y)
Supplement: Supplementary file 1 — Summary statistics of stable isotopic values of putative prey items for the threespine stickleback sampled in the brackish water Lake Engervann and marine areas. (PDF 176 kb) [file 12862_2018_1128_MOESM1_ESM.pdf]

Supplementary Table S1 Summary statistics of stable isotopic values of putative prey items for the threespine stickleback sampled in the brackish water Lake Engervann and marine areas.

Environment	Location	Site	Organism	$\delta^{13}C$	$\delta^{15}N$
Brackish water	Lake Engervann	Upper part	*Chironomida* spp.	-31.2	4.5
Brackish water	Lake Engervann	Middle part		-26.0	7.1
Brackish water	Lake Engervann	Lower part		-20.9	8.6
Brackish water	Lake Engervann	Upper part	*Gammarus* spp.	-27.6	7.1
Brackish water	Lake Engervann	Middle part		-23.1	8.0
Brackish water	Lake Engervann	Lower part		-22.2	7.9
Brackish water	Lake Engervann	Upper part	*Polychaeta* spp.	-27.6	8.3
Brackish water	Lake Engervann	Middle part		-24.4	9.9
Brackish water	Lake Engervann	Lower part		-19.3	11.4
Brackish water	Lake Engervann	Middle part	Shrimp spp.	-21.0	10.2
Brackish water	Lake Engervann	Lower part		-19.5	10.1
Brackish water	Lake Engervann	Lower part	Barnacle spp.	-21.2	9.9
Brackish water	Lake Engervann	Upper part	Snail1 spp.	-25.7	3.7
Marine benthic	Kalvøya Island	Shoreline	Shrimp1 spp.	-11.9	6.5
Marine benthic	Kalvøya Island	Shoreline	Shrimp2 spp.	-12.6	9.6
Marine benthic	Kalvøya Island	Shoreline	Shrimp3 spp.	-16.1	9.1
Marine benthic	Kalvøya Island	Shoreline	Snail spp2 spp.	-15.8	6.5
Marine benthic	Kalvøya Island	Shoreline	Blue mussel	-17.5	6.6
Marine pelagic	Steilene Island	Pelagic area	Shrimp spp.	-17.1	12.9
Marine pelagic	Steilene Island	Pelagic area	Shrimp spp.	-19.3	13.9
Marine pelagic	Steilene Island	Pelagic area	Shrimp spp.	-19.7	11.1
Marine pelagic	Steilene Island	Pelagic area	*Nudibrancha* spp.	-18.7	12.6
Marine pelagic	Steilene Island	Pelagic area	*Chaetognata* spp.	-18.6	14.2
Marine pelagic	Steilene Island	Pelagic area	*Copepoda* spp.	-19.1	14.1
