# Supplementary material for: Ongoing niche differentiation under high gene flow in a polymorphic brackish water threespine stickleback (Gasterosteus aculeatus) population
Source: BMC Evol Biol. 2018 Feb 5;18:14. doi: 10.1186/s12862-018-1128-y (PMC5800020; doi:10.1186/s12862-018-1128-y)
Supplement: Supplementary file 2 — STRUCTURE-HARVESTER results for the Lake Engervann sticklebacks. (PDF 640 kb) [file 12862_2018_1128_MOESM2_ESM.pdf]

Supplementary Table S2 STRUCTURE results for Lake Engervann threespine sticklebacks.

<u>The three additional analysis using neutral microsatellite loci and loci deviating from HWE:</u>

<u>Location (U/L)</u>:

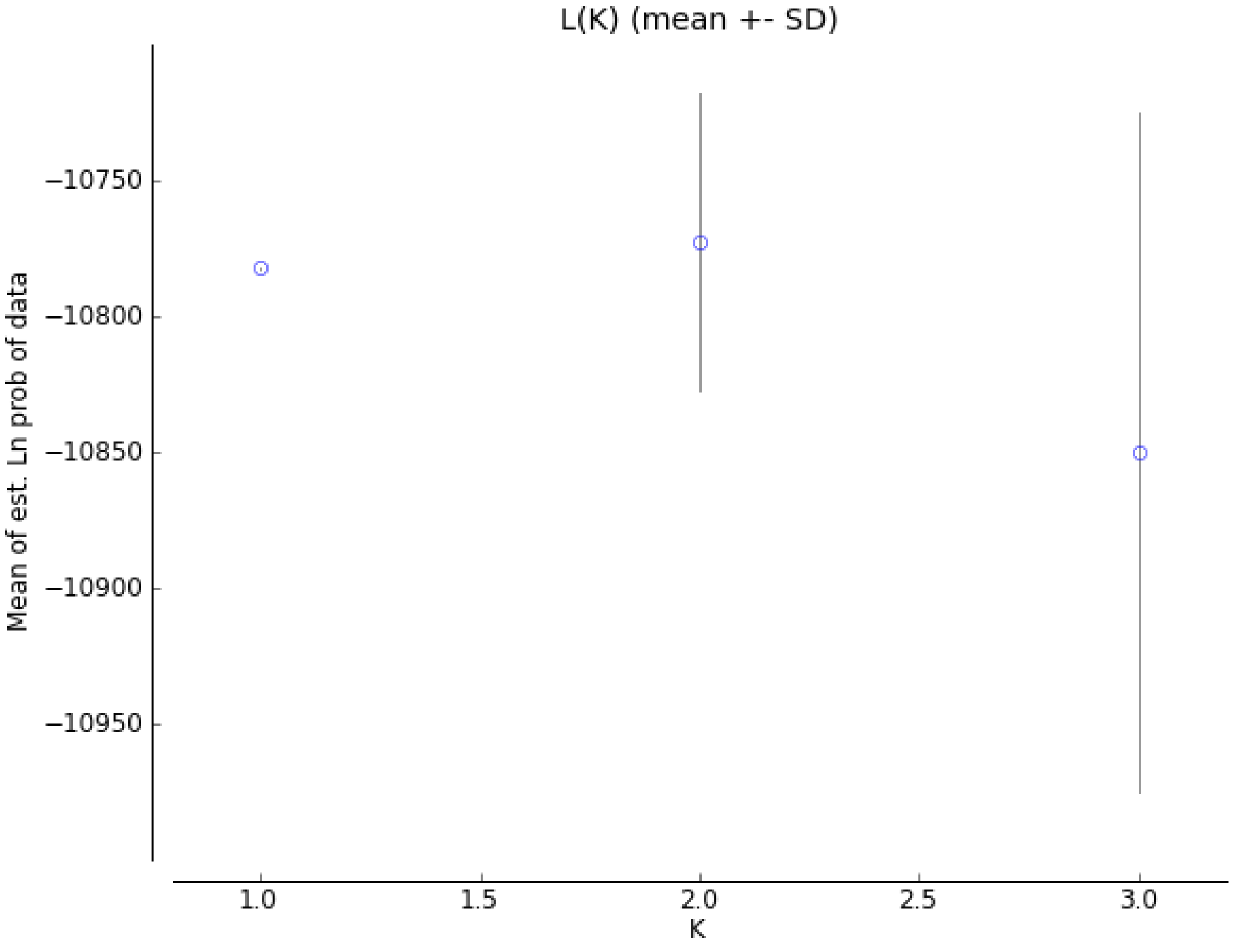

<u>K=2:</u>

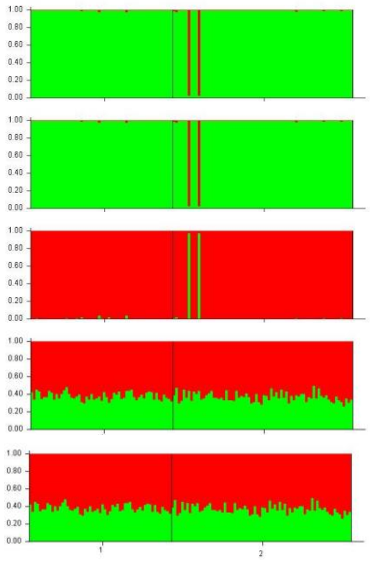

<u>K=3:</u>

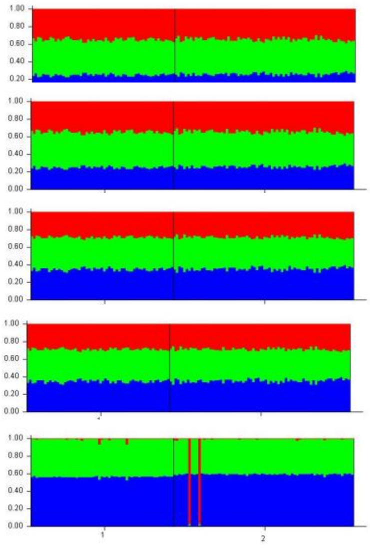

***Morph (CP/PP/LP)*:**

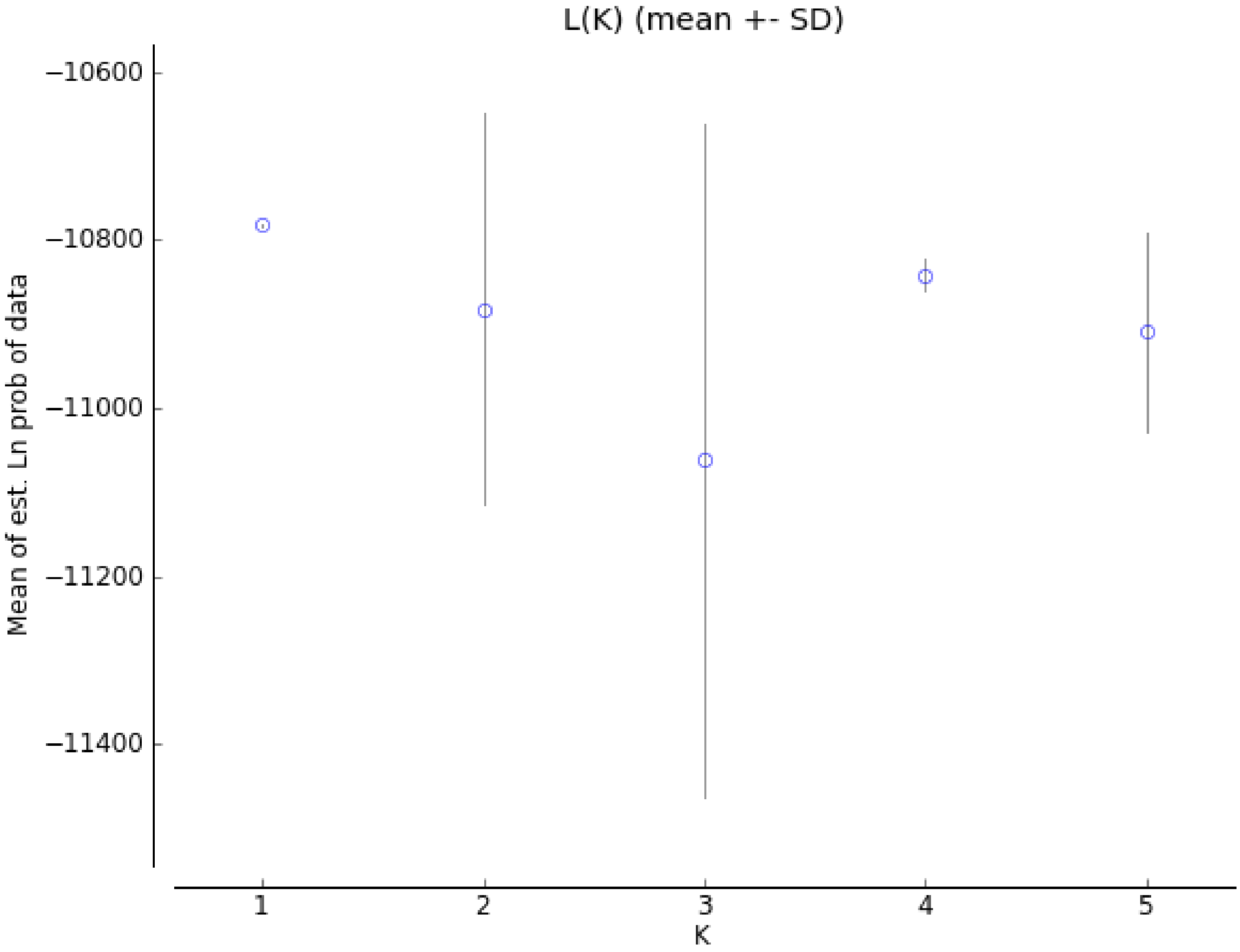

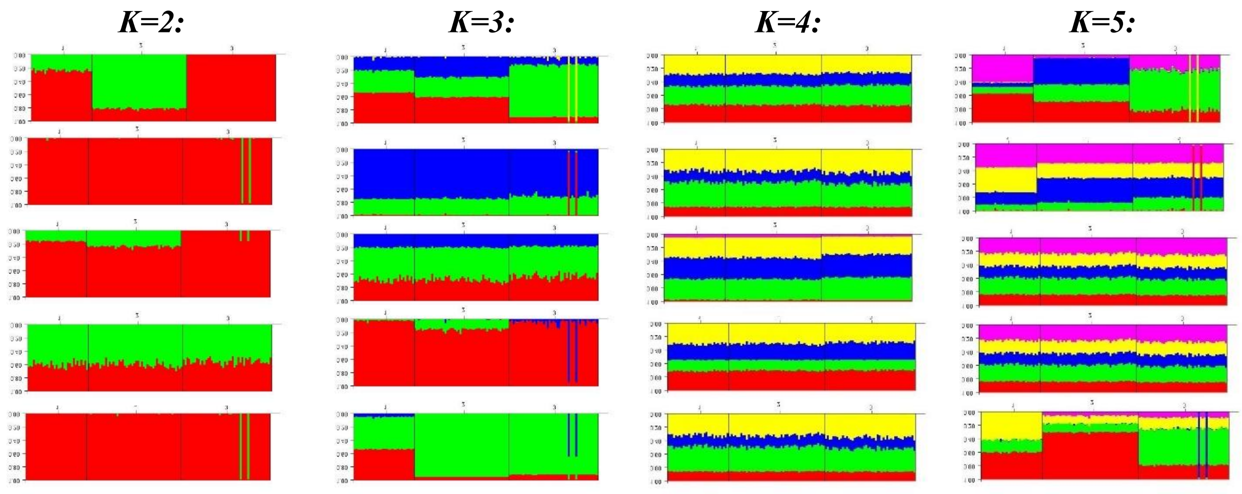

Location (U/L) x Morph (CP/PP/LP):

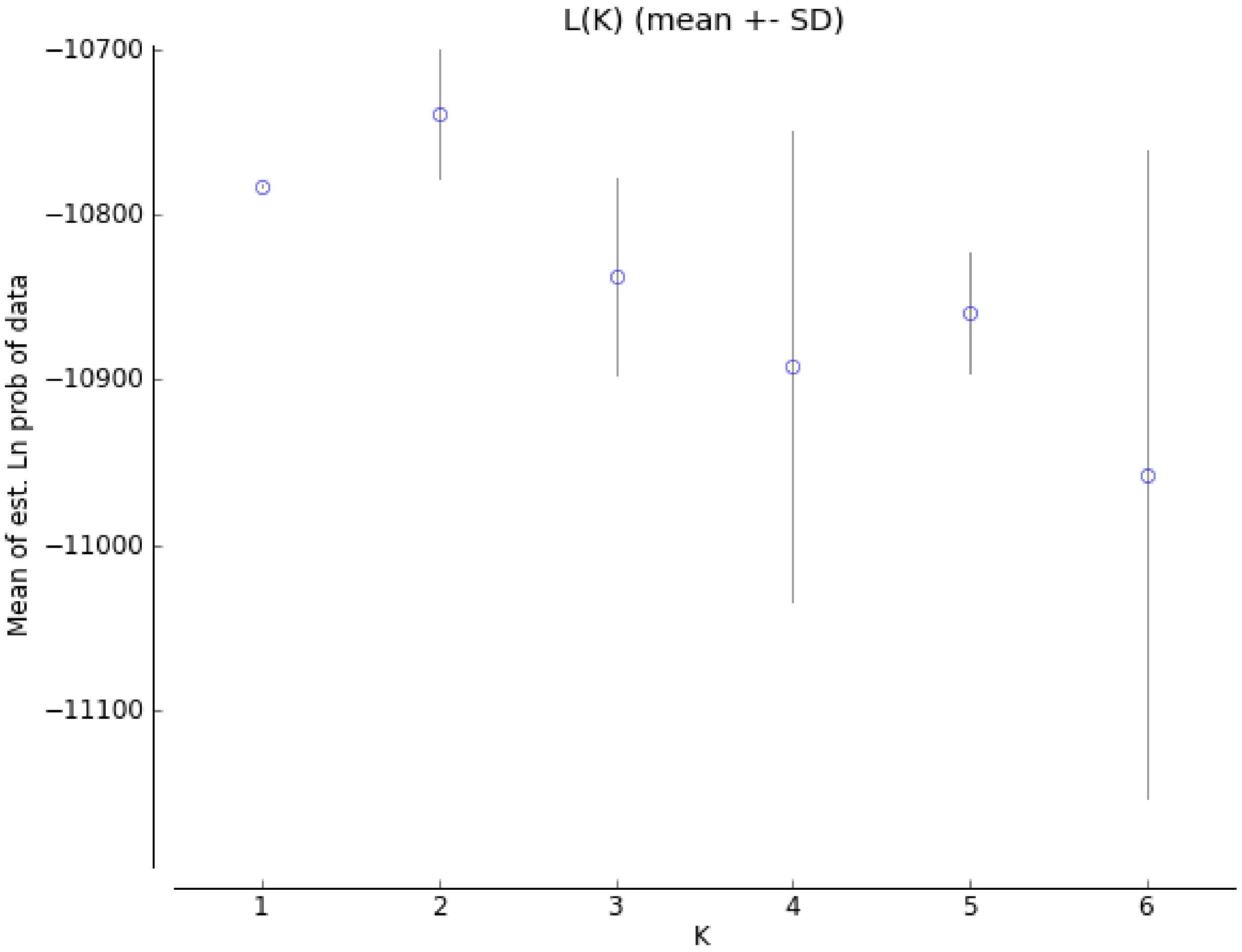

K=2: ***K=3:*** ***K=4:*** ***K=5:*** ***K=6:***

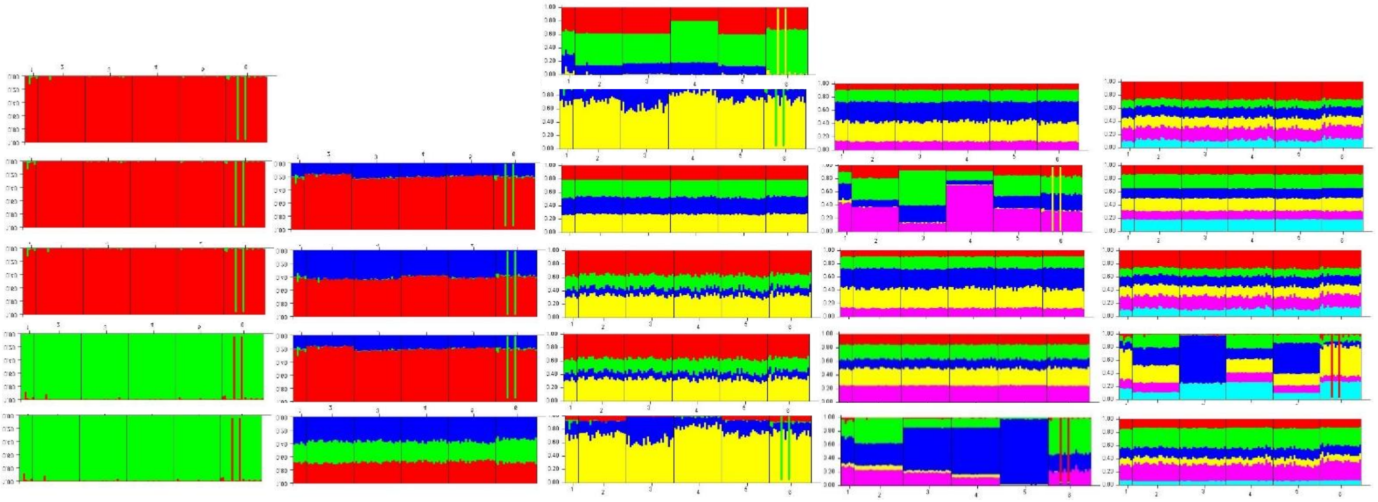

The original analysis using the 17 neutral microsatellite loci:

Morph (CP/PP/LP):

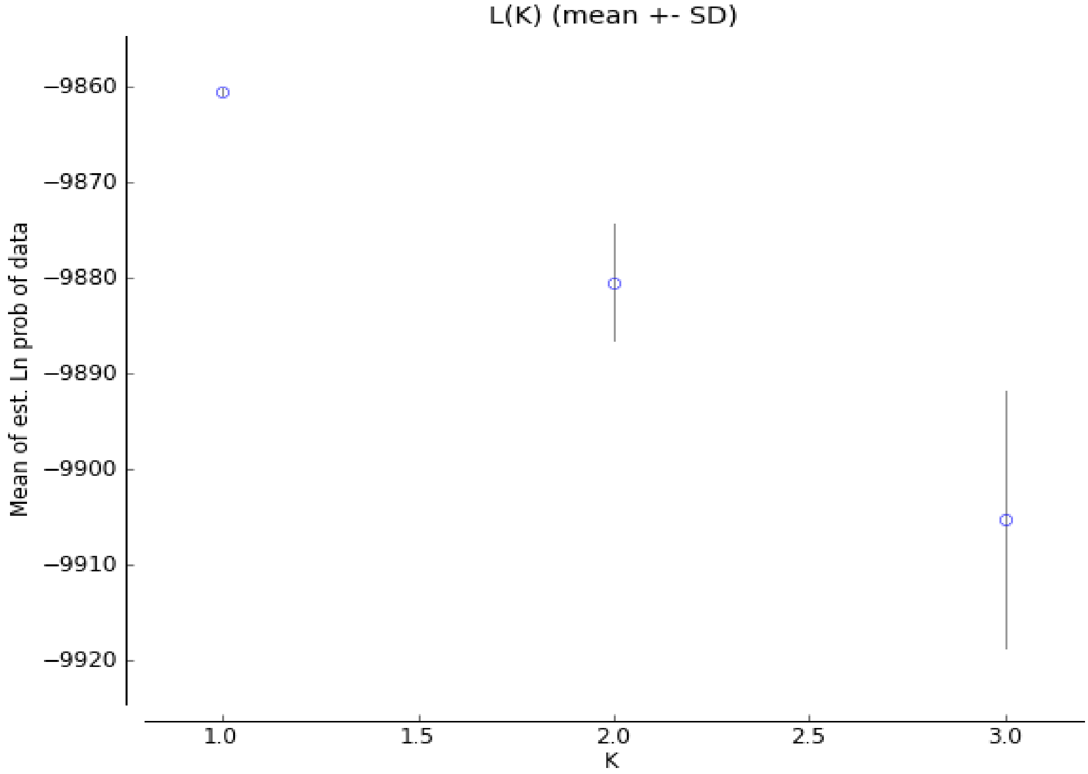

K=2:

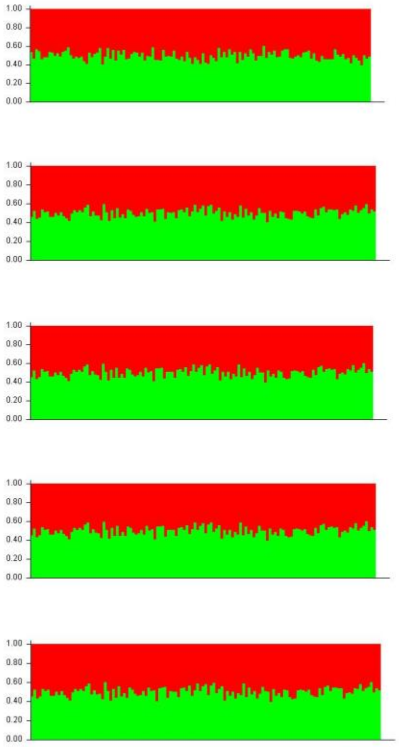

K=3:

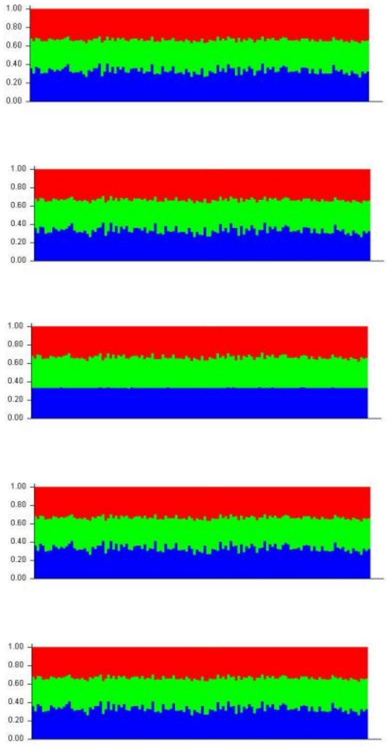

Run specifics:

The three additional analysis using neutral microsatellite loci and loci deviating from HWE:

Location (U/L)					
File name	Run #	K	Est. Ln prob. of data	Mean value of Ln likelihood	Variance of Ln likelihood
Locpri_run_3_f	3	1	-10782.5	-10704.8	155.5
Locpri_run_5_f	5	1	-10782.5	-10705.1	154.8
Locpri_run_4_f	4	1	-10783.5	-10705.2	156.4
Locpri_run_2_f	2	1	-10781.9	-10704.8	154.1
Locpri_run_1_f	1	1	-10782.7	-10705.1	155.1
Locpri_run_8_f	8	2	-10753.0	-10612.9	280.2
Locpri_run_7_f	7	2	-10741.8	-10613.4	256.8
Locpri_run_6_f	6	2	-10802.8	-10666.8	272.1
Locpri_run_9_f	9	2	-10715.0	-10612.8	204.3
Locpri_run_10_f	10	2	-10852.7	-10665.2	375.0
Locpri_run_14_f	14	3	-10795.9	-10674.5	242.9
Locpri_run_15_f	15	3	-11069.9	-10584.2	971.3
Locpri_run_11_f	11	3	-10828.1	-10603.7	448.8
Locpri_run_12_f	12	3	-10786.2	-10675.0	222.4
Locpri_run_13_f	13	3	-10770.3	-10605.7	329.2

Morph (CP/PP/LP)					
File name	Run #	K	Est. Ln prob. of data	Mean value of Ln likelihood	Variance of Ln likelihood
LocPri_run_1_f	1	1	-10782.8	-10704.6	156.3
LocPri_run_3_f	3	1	-10782.0	-10705.0	154.1
LocPri_run_2_f	2	1	-10782.2	-10705.0	154.4
LocPri_run_5_f	5	1	-10781.3	-10705.0	152.7
LocPri_run_4_f	4	1	-10780.0	-10705.0	149.9
LocPri_run_10_f	10	2	-10728.1	-10612.7	230.7
LocPri_run_7_f	7	2	-10890.7	-10685.6	410.2
LocPri_run_6_f	6	2	-10792.7	-10666.1	253.2
LocPri_run_9_f	9	2	-10716.6	-10613.0	207.4
LocPri_run_8_f	8	2	-11279.2	-10674.1	1210.1
LocPri_run_14_f	14	3	-10878.1	-10603.6	549.0
LocPri_run_12_f	12	3	-11021.2	-10597.7	846.9
LocPri_run_13_f	13	3	-10855.9	-10663.6	384.5
LocPri_run_11_f	11	3	-10789.8	-10671.3	236.9
LocPri_run_15_f	15	3	-11763.2	-10639.7	2247.0
LocPri_run_20_f	20	4	-10836.5	-10595.9	481.1

LocPri_run_18_f	18	4	-10854.7	-10672.1	365.1
LocPri_run_16_f	16	4	-10865.0	-10664.7	400.5
LocPri_run_19_f	19	4	-10818.5	-10672.3	292.5
LocPri_run_17_f	17	4	-10829.6	-10668.1	323.1
LocPri_run_21_f	21	5	-11076.4	-10579.5	993.8
LocPri_run_23_f	23	5	-10790.4	-10678.2	224.5
LocPri_run_22_f	22	5	-10984.5	-10586.5	796.0
LocPri_run_24_f	24	5	-10850.0	-10665.1	369.9
LocPri_run_25_f	25	5	-10845.7	-10589.6	512.2

Location (U/L) x Morph (CP/PP/LP)					
File name	Run #	K	Est. Ln prob. of data	Mean value of Ln likelihood	Variance of Ln likelihood
LocPri_run_5_f	5	1	-10782.9	-10705.5	154.8
LocPri_run_4_f	4	1	-10783.2	-10705.0	156.3
LocPri_run_3_f	3	1	-10783.2	-10704.9	156.7
LocPri_run_1_f	1	1	-10782.7	-10704.7	156.0
LocPri_run_2_f	2	1	-10782.6	-10704.9	155.3
LocPri_run_6_f	6	2	-10721.0	-10611.3	219.3
LocPri_run_8_f	8	2	-10808.9	-10661.4	295.0
LocPri_run_7_f	7	2	-10729.2	-10611.6	235.2
LocPri_run_9_f	9	2	-10713.2	-10610.4	205.8
LocPri_run_10_f	10	2	-10724.7	-10611.4	226.6
LocPri_run_12_f	12	3	-10803.4	-10668.2	270.4
LocPri_run_14_f	14	3	-10825.9	-10601.4	448.9
LocPri_run_15_f	15	3	-10864.5	-10592.9	543.0
LocPri_run_11_f	11	3	-10768.7	-10600.5	336.6
LocPri_run_13_f	13	3	-10924.0	-10592.0	664.0
LocPri_run_18_f	18	4	-11117.9	-10567.3	1101.2
LocPri_run_17_f	17	4	-10849.7	-10586.4	526.5
LocPri_run_16_f	16	4	-10868.2	-10665.0	406.5
LocPri_run_20_f	20	4	-10897.6	-10664.7	465.8
LocPri_run_19_f	19	4	-10725.2	-10609.2	232.1
LocPri_run_24_f	24	5	-10874.7	-10596.5	556.3
LocPri_run_21_f	21	5	-10821.9	-10595.9	451.9
LocPri_run_22_f	22	5	-10870.6	-10669.9	401.3
LocPri_run_23_f	23	5	-10905.6	-10661.6	487.9

LocPri_run_25_f	25	5	-10823.8	-10670.3	307.0
LocPri_run_26_f	26	6	-10824.1	-10583.8	480.5
LocPri_run_29_f	29	6	-11292.3	-10557.4	1469.9
LocPri_run_30_f	30	6	-10958.6	-10655.9	605.4
LocPri_run_28_f	28	6	-10820.9	-10673.1	295.4
LocPri_run_27_f	27	6	-10891.6	-10659.0	465.1

The original analysis using the 17 neutral microsatellite loci:

Morph (CP/PP/LP)					
File name	Run #	K	Est. Ln prob. of data	Mean value of Ln likelihood	Variance of Ln likelihood
Run_2_f	2	1	-9860.5	-9788.9	143.0
Run_4_f	4	1	-9861.1	-9789.0	144.1
Run_1_f	1	1	-9860.2	-9789.1	142.3
Run_3_f	3	1	-9860.2	-9788.9	142.4
Run_5_f	5	1	-9860.8	-9789.3	143.1
Run_9_f	9	2	-9889.2	-9757.0	264.3
Run_8_f	8	2	-9877.6	-9757.4	240.3
Run_10_f	10	2	-9884.6	-9756.9	155.4
Run_6_f	6	2	-9875.3	-9757.2	136.2
Run_7_f	7	2	-9875.8	-9757.6	136.5
Run_15_f	15	3	-9925.6	-9758.7	333.9
Run_11_f	11	3	-9894.7	-9758.7	272.0
Run_12_f	12	3	-9898.3	-9757.1	282.3
Run_13_f	13	3	-9912.6	-9753.6	318.0
Run_14_f	14	3	-9895.4	-9757.7	275.5
