# Supplementary material for: Ongoing niche differentiation under high gene flow in a polymorphic brackish water threespine stickleback (Gasterosteus aculeatus) population
Source: BMC Evol Biol. 2018 Feb 5;18:14. doi: 10.1186/s12862-018-1128-y (PMC5800020; doi:10.1186/s12862-018-1128-y)
Supplement: Supplementary file 3 — DAPC analyses (Dicriminant analysis of principal components) in adegenet (Jombart 2008) performed on the three lateral plate morphs of threespine stickleback from Lake Engervann. (PDF 299 kb) [file 12862_2018_1128_MOESM3_ESM.pdf]

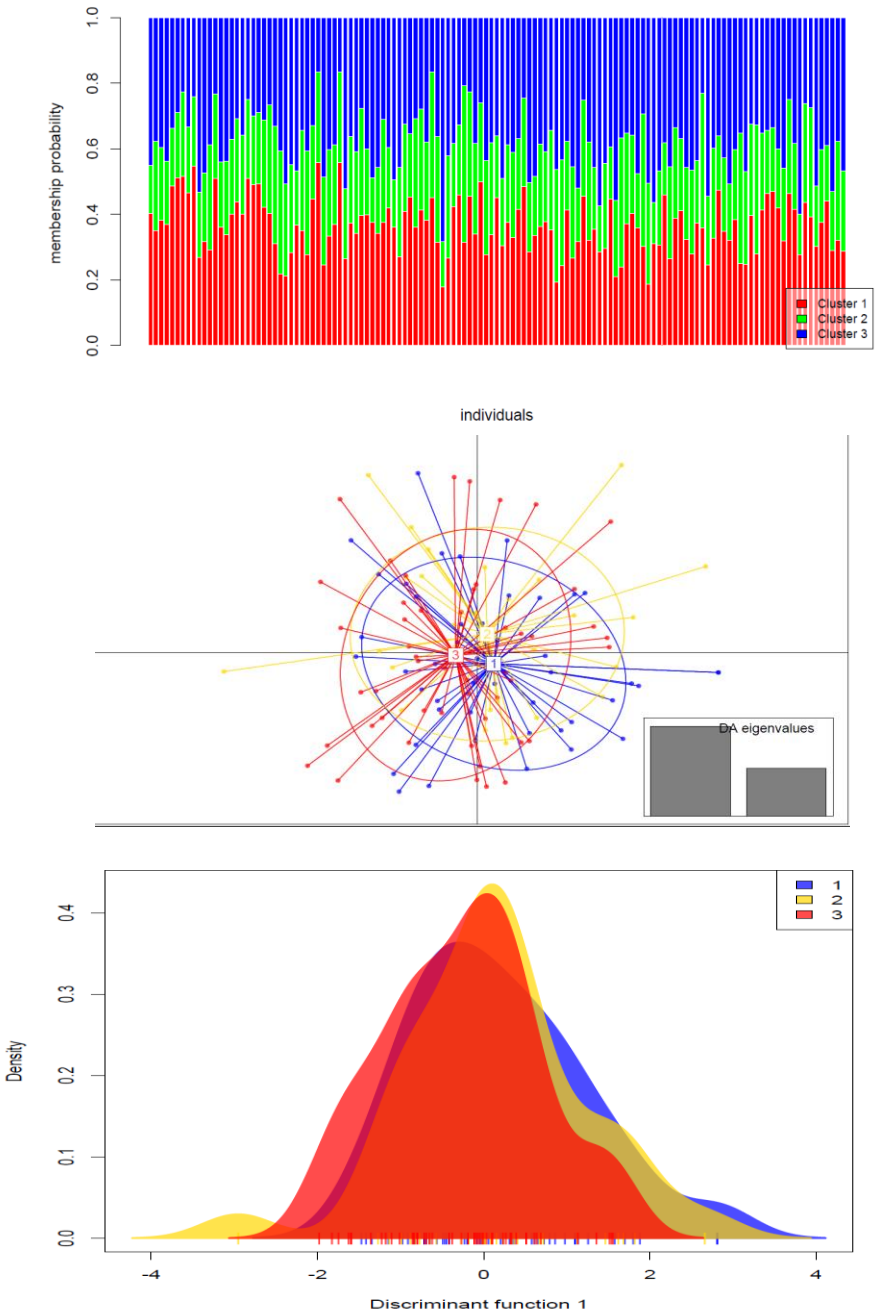

Supplementary Figure S3 DAPC analyses (Discriminant analysis of principal components) in *adegenet* (Jombart 2008) performed on the three lateral plate morphs of threespine stickleback from Lake Engervann; ***upper graph*** show "structure of plot assignment" and membership probability based on the first four PC axes recommended by the alpha score in the software adegenet (Jomabart 2008), ***middle graph*** show PCA axes 1 and 2, while the ***lower graph*** show discriminant function.

References:
Jombart T. adegenet: a R package for the multivariate analysis of genetic markers. Applications note. 2008; 24: 1403-1405.
