# Supplementary material for: Ongoing niche differentiation under high gene flow in a polymorphic brackish water threespine stickleback (Gasterosteus aculeatus) population
Source: BMC Evol Biol. 2018 Feb 5;18:14. doi: 10.1186/s12862-018-1128-y (PMC5800020; doi:10.1186/s12862-018-1128-y)
Supplement: Supplementary file 8 — The invertebrate fauna sampled on four locations (with an equal sampling effort performed on each locations) in the littoral area in the brackish water Lake Engervann on two dates (data report number of individuals sampled with the percentage contribution summed over two dates and combined for stations in the lower (Lower %) and upper sections (Upper %), and for the four sites and two dates combined in last column (Total %) (all data are taken from Halvorsen et al. [1]. Sampling sites 1 and 2 are from the lower and southern section on each side of the lake while site 3 and site 4 is situated on each side in upper northern part of the lake (see Fig. 1). (PDF 199 kb) [file 12862_2018_1128_MOESM8_ESM.pdf]

Supplementary Table S5 The invertebrate fauna sampled on four locations (with an equal sampling effort performed on each locations) in the littoral area in the brackish water Lake Engervann on two dates (data report number of individuals sampled with the percentage contribution summed over two dates and combined for stations in the lower (Lower %) and upper sections (Upper %), and for the four sites and two dates combined in last column (Total %) (all data are taken from Halvorsen et al. [1]. Sampling sites 1 and 2 are from the lower and southern section on each side of the lake while site 3 and site 4 is situated on each side in upper northern part of the lake (see Fig. 1).

	Date: 30.08.05				*Date*: 28.10.05				Lower %	Upper %	Total %
Sampling station:	*1*	*2*	*3*	*4*	*1*	*2*	*3*	*4*	*1 + 2*	*3 + 4*	*1, 2, 3, 4*
Chironomidae	45	334	318	382	1060	1320	558	3068	52.74	68.12	61.17
Oligochatea	414	281	160	666	368	220	150	300	24.53	20.09	22.09
Polychaeta	-	-	-	-	8	168	2	4	3.36	0.09	1.57
Hemiptera	-	3	-	-	-	-	-	-	0.06	0	0.03
Diptera larvae	15	11	36	27	8	-	4	8	0.65	1.18	0.94
Ceratopogonidae	2	12	6	12	-	24	-	-	0.73	0.28	0.48
Trichoptera (*Limnephilus* spp.)	-	2	-	1	4	-	8	8	0.11	0.27	0.20
Gastropoda (*Hydrobia ulvae*)	12	8	78	17	412	304	470	32	14.07	9.40	11.50
Planorbidae	-	-	-	-	-	4	-	-	0.08	0	0.03
Radix balthica	-	-	-	-	4	-	-	-	0.08	0	0.03
Bivalvia	-	-	-	1	-	-	-	-	0	0.02	0.01
Neomysis integer Leach	-	-	-	-	-	8	-	12	0.15	0.19	0.17
Gammarus duebeni Liljeborg	17	1	4	-	32	4	2	-	1.03	0.09	0.52
Palaemon adspersus Rathke	3	1	3	-	52	36	-	8	1.76	0.17	0.89
Zygoptera *	-	-	-	-	-	8	-	-	0.15	0	0.07
Tipulidae *	-	1	-	-	-	-	-	-	0.02	0	0.01
Coleoptera *	5	19	2	4	-	-	-	-	0.46	0.09	0.26
Oniscus asellus L. *	1	-	-	-	-	-	-	-	0.02	0	0.01

* These taxonomic groups represents land invertebrates.

References:

1. Halvorsen G, Often A, Svalastog D: Engervannet og Øverlandselva – statusrapport 2005. *NINA Minirapport* 136; 2005. (In Norwegian)
